# A new MMP‐mediated prodomain cleavage mechanism to activate bone morphogenetic proteins from the extracellular matrix

**DOI:** 10.1096/fj.202001264R

**Published:** 2021-02-25

**Authors:** Ariane G. Furlan, Chara E. S. Spanou, Alan R. F. Godwin, Alexander P. Wohl, Laura‐Marie A. Zimmermann, Thomas Imhof, Manuel Koch, Clair Baldock, Gerhard Sengle

**Affiliations:** ^1^ Center for Biochemistry Faculty of Medicine University Hospital Cologne University of Cologne Cologne Germany; ^2^ Department of Pediatrics and Adolescent Medicine Faculty of Medicine University Hospital Cologne University of Cologne Cologne Germany; ^3^ Wellcome Trust Centre for Cell‐Matrix Research School of Biological Sciences Faculty of Biology, Medicine and Health Manchester Academic Health Science Centre University of Manchester Manchester UK; ^4^ Division of Cell Matrix Biology and Regenerative Medicine School of Biological Sciences Faculty of Biology, Medicine and Health Manchester Academic Health Science Centre University of Manchester Manchester UK; ^5^ Institute for Dental Research and Oral Musculoskeletal Biology, Faculty of Medicine, University Hospital Cologne University of Cologne Cologne Germany; ^6^ Center for Molecular Medicine Cologne (CMMC) University of Cologne Cologne Germany; ^7^ Cologne Center for Musculoskeletal Biomechanics (CCMB) Cologne Germany

**Keywords:** bone morphogenetic protein (BMP), extracellular matrix (ECM), fibrillin, matrix metalloproteinase (MMP), single particle transmission electron microscopy

## Abstract

Since their discovery as pluripotent cytokines extractable from bone matrix, it has been speculated how bone morphogenetic proteins (BMPs) become released and activated from the extracellular matrix (ECM). In contrast to TGF‐βs, most investigated BMPs are secreted as bioactive prodomain (PD)–growth factor (GF) complexes (CPLXs). Recently, we demonstrated that PD‐dependent targeting of BMP‐7 CPLXs to the extracellular fibrillin microfibril (FMF) components fibrillin‐1 and ‐2 represents a BMP sequestration mechanism by rendering the GF latent. Understanding how BMPs become activated from ECM scaffolds such as FMF is crucial to elucidate pathomechanisms characterized by aberrant BMP activation and ECM destruction. Here, we describe a new MMP‐dependent BMP‐7 activation mechanism from ECM‐targeted pools via specific PD degradation. Using Edman sequencing and mutagenesis, we identified a new and conserved MMP‐13 cleavage site within the BMP‐7 PD. A degradation screen with different BMP family PDs and representative MMP family members suggested utilization of the identified site in a general MMP‐driven BMP activation mechanism. Furthermore, sandwich ELISA and solid phase cleavage studies in combination with bioactivity assays, single particle TEM, and in silico molecular docking experiments provided evidence that PD cleavage by MMP‐13 leads to BMP‐7 CPLX disintegration and bioactive GF release.

**Abbreviations**
BMP: bone morphogenetic protein
CD: circular dichroism spectroscopy
CPLX: complex
DPP: decapentaplegic
EBNA: Epstein‐Barr virus nuclear antigen
ECM: extracellular matrix
FMF: fibrillin microfibrils
FRET: fluorescence resonance energy transfer
GDF: growth and differentiation factor
GF: growth factor
HEK: human embryonic kidney
LAP: latency‐associated peptide
LLC: large latent complex
LTBP: latent TGF‐β‐binding protein
Mab: monoclonal antibody
MMP: matrix metalloproteinase
PD: prodomain
TEM: transmission electron microscopy
TGF: transforming growth factor
TLL2: tolloid‐like protein 2
TMB: tetramethylbenzidine

## 1 INTRODUCTION

Bone morphogenetic proteins (BMPs) belong to the TGF‐β superfamily of growth factors (GFs) and play a central role in a multitude of cellular processes during embryogenesis and postnatal homeostasis by guiding cell differentiation, proliferation, survival, and apoptosis.^1^, ^2^ Originally, BMPs were discovered as pluripotent cytokines extractable from bone matrix that are capable to induce ectopic bone formation.^3^ Further studies confirmed that BMPs are stored in embryonic and perinatal connective tissues such as kidney, skin, and blood vessels.^4^, ^5^, ^6^ These findings implicate that ECM‐bound BMPs serve an important function and that mechanisms for their utilization must exist to control their release at the appropriate time points when their action is required. For instance, the importance of ECM‐bound BMPs is not restricted to fetal osteogenesis but also for regeneration of adult bones as illustrated by limb‐specific *Bmp2* null mice which presented with irreversible spontaneous fractures.^7^

Among extracellular matrix (ECM) networks collagen fibers were first considered as the primary scaffold responsible for BMP sequestration.^8^, ^9^ Thereby, it was shown that procollagen‐2 is able to specifically bind BMP‐2 and thereby influencing its bioactivity.^10^ In another prominent example it was found that collagen IV controls the bioavailability of the BMP homolog decapentaplegic (dpp) in drosophila by binding dpp directly or promoting the interaction with its receptor complex.^11^

Our previous work identified highly specific interactions between the prodomains (PDs) of BMPs and the extracellular glycoproteins fibrillin‐1 and ‐2.^6^, ^12^ In addition, extracellular co‐immunostaining of BMPs and fibrillin‐1 suggested that fibrillin‐1 microfibrils serve as storage platforms for these GFs.^4^, ^5^, ^6^ Fibrillins are 350 kDa glycoproteins with a conserved multidomain structure. In tissues, fibrillin‐1 and ‐2 monomers are arranged into supramolecular, beads‐on‐a‐string fibrillin microfibrils (FMF) with a diameter of 10‐12 nm.^13^ The importance of FMF integrity becomes evident in congenital connective tissue disorders, caused by mutations in the fibrillin‐1 and ‐2 coding genes (*FBN1* and *FBN2*), the so‐called fibrillinopathies.^14^ The fibrillinopathies represent disorders with similar, but also opposing clinical features affecting the musculoskeletal, cardiovascular, ocular, pulmonary, and dermal system.^15^ From these clinical features it can be concluded that fibrillins modulate GF‐driven growth and differentiation processes in connective tissues. Also, analysis of fibrillin deficient mouse models corroborated this notion. An accelerated maturation of *Fbn1^‐/‐^* null osteoblasts was detected due to increased availability of non‐ECM‐targeted BMPs.^16^ Furthermore, genetic ablation of fibrillin‐2 resulted in limb patterning and muscle maturation defects caused by dysregulated BMP signaling.^17^, ^18^ Meanwhile it is an established concept that FMF target and sequester TGF‐β superfamily GFs and thereby regulate their bioavailability.^19^, ^20^

TGF‐β depends on latent TGF‐β‐binding proteins (LTBPs) for sufficient secretion and targeting to the ECM in the form of a large latent TGF‐β complex (LLC) formed by cysteine bridges of its PD to specialized 8‐cysteine LTBP domains.^21^, ^22^, ^23^ Once secreted, the LLC is targeted to the ECM via LTBP binding to FMF and fibronectin.^24^, ^25^, ^26^, ^27^ The mechanisms of TGF‐β activation from FMF‐bound pools, either by mechanical activation through integrin αv binding to the TGF‐β PD (also known as latency‐associated peptide: LAP),^28^, ^29^, ^30^ or upon metalloproteinase cleavage of LAP^31^ have been described. The concept that proteolytic cleavage of LAP leads to the activation of TGF‐β‐1 GF has been explored in in vitro experiments using MMP‐2 and MMP‐9.^32^ Also a combined activation mechanism has been described, where αvβ8 integrin binds to TGF‐β‐1 via LAP and enables MMP‐14 cleavage of LAP and release of TGF‐β‐1 GF.^33^ Another TGF‐β‐1 GF activation model proposed that BMP‐1 cleavage of LTBP‐1 at N‐ and C‐terminal sites releases truncated LLC from the ECM which is followed by a second MMP‐2‐mediated cleavage event of LAP.^34^ GDF‐8 and GDF‐11 are activated by BMP‐1/Tolloid (TLD) metalloprotease‐mediated cleavage of the PD.^35^, ^36^ Recently, tolloid‐like protein 2 (TLL2) was also shown to cleave the PD of GDF‐8 and thereby activate it from the latent state.^37^ Similarly, our previous in vitro results predicted a similar mechanism for BMP‐10.^12^ Although much work has been undertaken to investigate the mechanisms of TGF‐β activation, the regulatory pathways of other TGF‐β superfamily members such as BMPs remain largely unknown.

In contrast to TGF‐β, most BMPs are secreted as bioactive PD‐GF complexes (CPLXs) to the extracellular space.^12^, ^38^, ^39^, ^40^, ^41^ Previously, we showed that the BMP‐7 PD does not confer latency to the GF in solution, and that BMP type II receptors have free access to their binding sites on the GF. This suggestion arose from velocity sedimentation experiments in sucrose gradients showing that BMP type II receptor binding to the GF results in release of the free PD as a dimer from the CPLX.^39^ Recently, we could show that upon binding of the BMP‐7 PD to the fibrillin‐1N‐terminal unique domain, a conformational change in the BMP‐7 CPLX is induced which renders the GF inactive by locking the α2‐helix of the PD in place, denying access to the BMP type II receptor site.^42^

However, little is known about how BMPs are released and activated once they are targeted to the ECM. Therefore, the aim of this study was to investigate new BMP activation mechanisms from FMF‐targeted pools.

## 2 MATERIALS AND METHODS

### 2.1 Ethics statement

This study was carried out in strict accordance with German federal law on animal welfare, and the protocols were approved by the “Landesamt für Natur, Umwelt und Verbraucherschutz Nordrhein‐Westfalen” (permit no. 84‐02.04.2014.A397 for breeding and permit No. 84‐02.05.40.14.115 for euthanasia).

### 2.2 Antibodies

Previously described monoclonal anti‐BMP‐7 PD antibodies mab2 and mab33^4^ were kindly provided by Dr Lynn Sakai (Oregon Health and Science University). For western blots, mab33 was either used alone (1:1000 dilution), or in a mixture (1:1 molar ratio) together with mab2 (1:1000 dilution). The generation of polyclonal anti‐fibrillin‐1 antibody was previouly described.^43^ Polyclonal antibody against BMP‐7 GF was purchased from PeproTech (#500‐P198, Rocky Hill, NJ).

### 2.3 Cell culture

Primary murine skin fibroblasts were isolated from newborn mice.^44^ Primary dermal fibroblasts and HEK 293 cells were cultured in Dulbecco's Modified Eagle's medium (DMEM GlutaMAX, Invitrogen, Carlsbad, CA) supplemented with 10% of fetal bovine serum and penicillin/streptomycin.

### 2.4 mRNA expression analysis via quantitative real‐time PCR

A total of 1 × 10^5^ HEK 293 cells or primary murine skin fibroblasts cells were grown in 6‐well plates prior to BMP GF stimulation at 100 ng/mL. After 24 hours of BMP stimulation, RNA extraction was performed by adding 1 mL of TRIzol Reagent (Thermo Fisher Scientific, Waltham, MA) according to the manufacturer's protocol. A subsequent sample purification step was included using the RNeasy kit (Qiagen, Venlo, The Netherlands), and residual DNA contamination was removed from each sample using the Turbo DNA‐free kit (Ambion, Austin, TX). RNA samples were quantified by photospectrometry, and 1.0 μg of RNA per sample was reverse‐transcribed using the Bio‐Rad iScript cDNA synthesis kit (Bio‐Rad, Hercules, CA). Quantitative PCR was performed using SensiFAST SYBR Hi‐ROX Kit in 25 µL reaction volume (Meridian Bioscience, Cincinnati, OH). PCR was conducted with the StepOnePlus system (Applied Biosystems, Thermo Fisher Scientific). The standard annealing temperature of 60°C was chosen for the selected primer pairs (*Mmp2F:* CAAGTTCCCCGGCGATGTC, *Mmp2R:* TTCTGGTCAAGGTCACCTGTC; *Mmp3F:* ACATGGAGACTTTGTCCCTTTTG, *Mmp3R:* TTGGCTGAGTGGTAGAGTCCC; *Mmp13F:* TGTTTGCAGAGCACTACTTGAA, *Mmp13R:* CAGTCACCTCTAAGCCAAAGAAA). Analysis of data was performed using the 2^−ΔΔCt^ method^45^ and quantitated relative to the murine *Arbp* or human *GAPDH* gene. Gene expression was normalized to BSA‐treated control samples, which provided an arbitrary constant for comparative fold expression. Primer pairs for human *MMP* genes were purchased from Qiagen.

### 2.5 Protein expression and purification

BMP‐7 CPLX was expressed and purified as described before.^4^ Briefly, the HEK 293 EBNA cell line stably transfected with N‐terminally His_6_‐tagged BMP‐7 CPLX was kindly provided by Dr Lynn Sakai (Oregon Health and Science University). Cells were maintained in triple flasks, medium was collected, and affinity purified via nickel chelate affinity chromatography using the Ni‐NTA resin (Cube Biotech, Germany). The highest purity fractions of BMP‐7 CPLX were eluted with imidazole at a concentration of 50‐250 mM. PDs of BMP‐4, ‐5, ‐7, and ‐10 were expressed in *E. coli* and purified as previously described.^6^, ^12^ cDNAs encoding for BMP‐7 PD mutant variants, PDs of human BMP‐9 (K^23^‐R^319^), human TGF‐β‐1 (L^30^‐R^279^), and TGF‐β‐2 (L^21^‐R^330^) were generated by gene synthesis (Genewiz, South Plainfield, NJ), cloned into the pET11a vector, overexpressed in *E coli* with a C‐terminally placed His_6_‐tag, and purified via Ni‐NTA.^6^, ^46^ The murine proMMP‐2, ‐7, ‐8, ‐9, and ‐13 (MMP2: NP_032636.1, aa A^30^‐C^662^; MMP7: NP_034940.2 aa L^21^‐L^267^, MMP8: NP_032637.3, aa F^21^‐S^465^; MMP9: NP_038627.1, aa A^20^‐P^730^; MMP13: NP_032633.1, L^19^‐C^472^) were expressed and purified as described previously.^47^ MMP‐3, MMP‐12, GDF‐8 PD, and BMP‐7 GF were purchased from R&D Systems (Minneapolis, MN).

### 2.6 Proteolytic cleavage assays

MMPs were activated with 250 µM of amino‐phenyl mercuric acetate (APMA) (Sigma‐Aldrich, St. Louis, MO) for 2 hours at 37°C. For buffer exchange of solubilized BMP PDs to TC buffer (50 mM of Tris‐HCl pH 7.5 and 1 mM of CaCl_2_), Amicon ultra 0.5 mL centrifugal filters (Merck Millipore, Burlington, MA) were used. A total of 10 nM of each activated MMP was incubated with 1 µM of BMP PD in 50 µL for 2 hours at 25°C. Fragments were analyzed by western blotting and silver staining. For Edman sequencing, 6 µg of BMP‐7 PDs were incubated with 60 ng of MMP‐2, MMP‐3, or MMP‐13. Fragments were separated by 10%‐20% SDS‐PAGE and transferred to a PVDF membrane. After staining with Ponceau S, the cleavage products were excised, and subjected to N‐terminal Edman degradation performed by Proteome Factory AG (Berlin, Germany). MMP activity was assessed through incubation with a quenched Omni‐MMP fluorogenic substrate (#BML‐P126‐0001, Enzo Life Sciences, Lörrach, Germany) in black 96‐well plates (Thermo Fisher Scientific, Waltham, MA). Cleavage of the fluorescence resonance energy transfer (FRET)‐based substrate (acceptor: MCA, donor: Dpa), led to fluorescence at 400 nm. For each assay, 2 µM of MMP substrate was incubated with 10 nM of the respective MMP in 100 µL of TC buffer for 30 minutes, at 25°C, followed by detection of fluorescence emission at 400 nm by an Infinite M1000 spectral photometer (Tecan, Switzerland).

### 2.7 Circular dichroism spectroscopy

BMP‐7 PD variants were dialyzed into 5 mM of HClO_4_. CD spectra were recorded using a Jasco J‐715 spectropolarimeter at 260‐170 nm in a 0.1‐mm path length quartz cell (Hellma, Germany) at 20°C. After subtraction of the buffer contribution, data were converted to Δϵ.

### 2.8 ELISA and sandwich ELISA

For ELISA, 100 ng/mL of BMP‐7 CPLX was coated to microtiter plates (Nalge Nunc, Rochester, NY) in PBS overnight at 4°C. Wells were blocked with 5% nonfat dry milk/TBS for 1 hour at RT and washed three times with 0.025% TBS‐tween, afterwards. Directly coated BMP‐7 CPLX was incubated with MMPs at a molar ratio of 1:100 (MMP:BMP‐7 CPLX) for 2 hours at RT in TC buffer. For sandwich ELISA detection, BMP‐7 CPLX after MMP‐13 cleavage was transferred to anti‐BMP‐7 GF antibody coated wells (10 µg/mL, PeproTech) and incubated for 1 hour. Wells were washed three times with 0.025% TBS‐tween and incubated with detection antibody against BMP‐7 PD (mab33 at 1:1000 dilution) in 2.5% nonfat dry milk/TBS for 2 hours at RT, followed by 1 hour incubation of HRP‐conjugated anti‐rabbit antibody in 2.5% nonfat dry milk/TBS at RT. Subsequently, wells were washed three times with TBS‐tween, and incubated with 50 µL of 1‐Step Ultra TMB‐ELISA substrate solution for signal development (Thermo Fisher Scientific, Waltham, MA). OD was read at 450 nm using a Sunrise microplate reader (Tecan).

### 2.9 MMP‐13 cleavage assays on solid phase

For the generation of an assembled ECM fiber network, 1 × 10^6^ mouse skin fibroblasts were seeded on 6‐well plates and cultivated for 4 days, followed by cell removal using deoxycholate.^48^ In brief, cell cultures were washed once with PBS and then, treated twice with 0.5% sodium deoxycholate in 10 mM of Tris‐HCl buffer, pH 8.0, at 0°C for 10 minutes. The plates were then allowed to dry overnight at RT. Subsequently, wells were blocked in 5% BSA followed by incubation with BMP‐7 CPLX. To assess co‐localization between added BMP‐7 CPLX and fibrillin‐1 fibers by immunofluorescence, cells were grown on 24‐well plates. To demonstrate a direct interaction between added BMP‐7 CPLX and assembled ECM fibers by ELISA‐style solid phase interaction assay, mouse fibroblasts were grown on 96‐well plates. For this, 100 ng/mL of BMP‐7 CPLX was titrated onto ECM‐coated dishes following a 1:2 serial dilution in TBS buffer containing 1% of BSA at RT for 2 hours. BMP‐7 CPLX immobilized to ECM fibers was submitted to MMP‐13 cleavage (50 ng/mL) in 1 mL of TC buffer for 2 hours at 37°C. Afterwards, the supernatant was collected and subjected to TCA precipitation for western blot analysis or lyophilized to be subjected to BMP bioactivity assays.

### 2.10 BMP bioactivity assay

Supernatant from 6‐well plates containing GF released from ECM‐targeted BMP‐7 CPLX after MMP‐13 cleavage was collected and dialyzed in mini dialysis tubes with a molecular weight cut‐off of 2 kDa into 100 mM acetic acid overnight at 4°C. After dialysis, samples were shock‐frozen in liquid nitrogen and lyophilized overnight. Subsequently, samples were resuspended in 10 µL of 4 mM HCl and administered to BMP bioactivity assays. To measure BMP bioactivity murine C2C12 myoblasts were utilized as reporter cell line. For each measurement, 3.5 × 10^4^ cells/well were seeded onto 96‐well plates. Stimulation was performed in eight wells per concentration in triplicates. Two or three microliters of the obtained supernatant after MMP‐13 cleavage of ECM‐bound BMP‐7 CPLX and 2 or 3 µL of the supernatant without MMP‐13 incubation were added for C2C12 cell stimulation. After 5 hours, the total mRNA content of cells was harvested, reverse‐transcribed, and subjected to qPCR to measure the mRNA levels of BMP response gene *Id3* (inhibitor of differentiation 3).^39^ *Id3* mRNA levels were normalized to the mRNA expression of *ARBP* (“acidic ribosomal binding protein”) which served as housekeeping gene. A total of 10 ng/mL of BMP‐7 GF (R&D systems) was added to the medium as positive control, and incubation of cells with 0.1% of BSA served as untreated negative control.

### 2.11 Transmission electron microscopy (TEM) and single particle analysis

BMP‐7 CPLX alone, after dialysis into 1 M urea, or after incubation with MMP‐13 for 2 hours was negatively stained as described previously.^49^ BMP‐7 CPLX alone and cleaved with MMP‐13 data were recorded at ×30 000 magnification using a FEI Tecnai 12 twin TEM at 120 kV using a Tietz TVIPs F214A CCD camera. Images were recorded with a 1‐s exposure at defocus values of −0.5 to −1.6 μm at 1.5 Å/pixel (Figure 9C). BMP‐7 CPLX after dialysis into 1 M urea data were collected on a FEI Tecnai G2 Polara TEM operating at 300 kV equipped with a Gatan K2 summit direct detector. Images were recorded at ×23 000 magnification with a 1‐s exposure in integrating mode at defocus values of −0.5 to −1.6 μm at 1.67 Å/pixel. Single particle analysis was performed using Relion.^50^, ^51^ Particles were selected by a combination of manual and automated picking. The total number of particles selected for BMP‐7 CPLX either alone, after MMP‐13 incubation, or in the presence of 1 M urea was approximately 900, 6770, or 9600, respectively. Each data set was subjected to two‐dimensional classification.

### 2.12 Dynamic light scattering

BMP‐7 CPLX was dialyzed overnight into TC buffer and cleaved with MMP‐13 as described above. DLS measurements of the cleaved and non‐cleaved control BMP‐7 CPLX were then taken using a Zetasizer Nano‐S (Malvern, Herfordshire, UK) at a controlled temperature of 25°C.

### 2.13 Molecular docking experiments

The generation of the BMP‐7 CPLX closed‐ring shape model (Figure 6), based on the TGF‐β‐1 LAP crystal structure (Protein Data Bank code 3RJR) as a template, with MODELLER^52^ in UCSF Chimera^53^ was as described.^42^ In this model, a break in the peptide chain was introduced at residue Pro^80^ to allow the PD to be rotated into an open conformation without moving the N‐terminal region. Next, using the Chimera software a peptide bond with a phi torsional angle of −60° was introduced at the exact same position in order to re‐join the polypeptide chains without moving the N‐terminal PD region. The BMP‐9 CPLX structure (Protein Data Bank code 4YCI) and the BMP‐7 CPLX EM map were used to guide rotation of the PD into an open conformation. To obtain a structural model of BMP‐7 PD in the open V‐shape conformation, BMP‐7 PD was modeled on the proactivin CPLX structure (Protein Data Bank code 5HLZ) using Swiss‐model. To gain structural insight into the MMP‐13 cleavage mechanism, BMP‐7 PD in the open conformation or the closed‐ring BMP‐7 CPLX, inputted as the “receptors” were docked to the crystal structure of the activated MMP‐13 (Protein Data Bank code 4fu4) inputted as the “ligand” in the ClusPro2.0 server^54^ after deleting the structure of the co‐crystallized peptide using UCSF chimera software. Subsequently, 30 molecular docking models were screened for each in silico experiment and models 21 and 16 were selected for closed and open BMP‐7 PD conformations, respectively, due to excellent alignment of the MMP‐13 catalytic site (His^222^, His^226^, and His^232^) to the prime region ^83^MLD^85^ of the cleavage site. To obtain a theoretical model of MMP‐13 cleaving the BMP‐7 CPLX closed‐ring, model 21 was structurally aligned to each BMP‐7 PD monomer of the closed BMP‐7 CPLX model at the ^83^MLD^85^ site. Images were taken both in ribbon and surface representations. To pinpoint the exact molecular requirements for cleavage in the open BMP‐7 PD conformation, model 16 was superimposed to the activated MMP‐13 structure (Protein Data Bank code 4fu4) and the positioning of the co‐crystallized peptide was compared to the ^83^MLD^85^ site of BMP‐7 PD. Ions and metals were visualized using the 4fu4 template and images were taken in ribbon representation. To generate the open V‐shape BMP‐7 CPLX model, BMP‐7 PD was assembled into a dimer using Swiss‐model and the proactivin CPLX was structurally aligned to the BMP‐7 PD dimer. Next, the monomers of the BMP‐7 GF crystal structure (Protein Data Bank code 1LX5) were structurally aligned to the monomers of the proactivin GF after deleting the structure of the ActRII extracellular domain using UCSF chimera. To understand how MMP‐13 is cleaving the open V‐shape BMP‐7 CPLX, model 16 was structurally aligned to one BMP‐7 PD of the CPLX and images were taken both in ribbon and surface representations.

### 2.14 Statistical analysis

Data are expressed as mean ± SD. Statistical analyses were performed using GraphPad Prism software and the significance of differences between groups was determined by applying an unpaired two‐tailed Student's test. Values of *P* ≤ .05 were considered significant.

## 3 RESULTS

### 3.1 BMP‐7 GF stimulates metalloproteinase activity leading to specific cleavage of BMP‐7 PD

**FIGURE 1:**
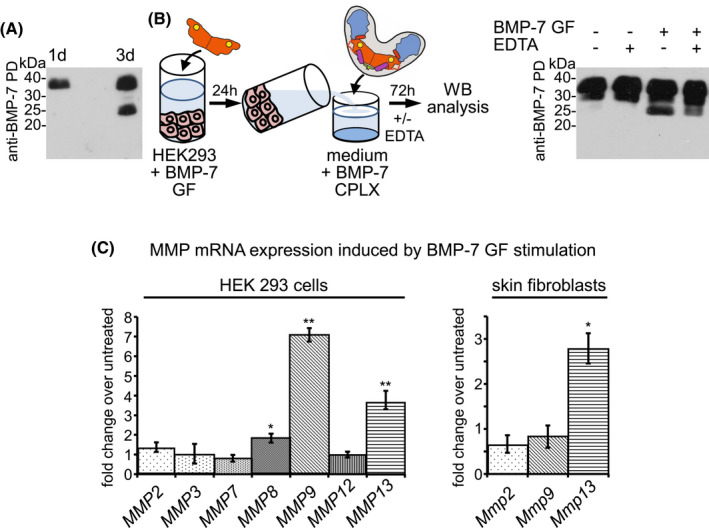
Specific cleavage of BMP‐7 PD in cell culture medium. A, Cell culture supernatant from HEK 293 cells transiently transfected with BMP‐7 cDNA coding for BMP‐7 CPLX was monitored via western blot analysis for PD integrity using antibody against BMP‐7 PD (mab2/mab33, 1:1 mixture) after 1 and 3 days (d). B, (left) Scheme illustrating experimental design: HEK 293 cells were stimulated with BMP‐7 GF only (100 ng/mL), and the cell culture supernatant was collected after 24 h. Subsequently, the medium was incubated with recombinant BMP‐7 CPLX protein for 72 h in the presence or absence of 10 mM EDTA, followed by western blot analysis using anti‐BMP‐7 PD antibody (mab2/mab33, 1:1 mixture). C, mRNA expression of MMPs in HEK 293 cells and primary murine dermal fibroblasts measured by qPCR after 24 h of BMP‐7 GF stimulation. Error bars show standard deviation of three independent experiments (N = 3). Statistical analysis was by an unpaired two‐tailed Student's t test. **P ≤ .01, *P ≤ .05

Western blot analysis of HEK 293 cells transiently transfected with full length BMP‐7 cDNA encoding for the entire BMP‐7 PD‐GF CPLX showed two bands for BMP‐7 PD after 3 days of culture. After 1 day of transfection, only the expected size of full length BMP‐7 PD at approximately 37 kDa was detected, while at day 3 an additional band of about 25 kDa could be observed (Figure 1A). This suggested that after secretion a specific proteolytic event occurred within the BMP‐7 PD. To dissect whether BMP‐7 PD cleavage may have been initiated by BMP autostimulation or by accumulation of general protease activity in the culture media over time, HEK 293 cells were incubated with BMP‐7 GF only for 24 hours and the resulting supernatant was incubated with recombinantly expressed and affinity‐purified BMP‐7 CPLX protein (Figure 1B). Results from western blot analysis showed that only supernatant obtained from BMP‐7 GF stimulated cell layers caused specific BMP‐7 PD cleavage. This cleavage was inhibited upon addition of EDTA, suggesting that BMP‐7 PD cleavage was caused by metalloproteinase activity (Figure 1B). Based on the findings of previous reports, we hypothesized that BMP‐7 stimulation of HEK 293 cells may have induced upregulation of MMPs which in turn led to PD degradation of the BMP‐7 CPLX. To test this assumption, we measured the mRNA levels of representatives of MMP family subgroups after 24 hours of HEK 293 cell stimulation with BMP‐7 GF (Figure 1C). We found that BMP‐7 GF administration induced a significant upregulation of MMP‐8, ‐9, and ‐13 mRNA expression suggesting that the BMP‐7 PD was degraded by a BMP‐induced MMP activity. Stimulation of ECM resident cells such as skin fibroblasts confirmed that BMP‐7 GF is able to induce most robustly MMP‐13 mRNA expression (Figure 1C). These experiments suggested that BMP‐7 GF is capable to stimulate metalloproteinase expression which in turn leads to specific cleavage of BMP‐7 PD.

### 3.2 BMP‐7 PD as new substrate for MMPs

**FIGURE 2:**
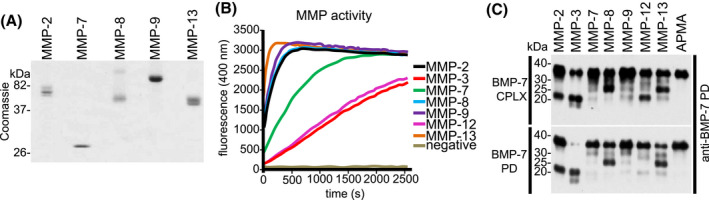
Proteolytic processing of BMP‐7 PD by representatives of MMP family subgroups. A, Assessment of integrity and purity of recombinantly expressed and affinity‐purified MMPs by SDS‐PAGE (10% gel) and Coomassie staining. B, Measurement of MMP activity was assessed by fluorescence detected after incubation with a FRET peptide substrate. C, BMP‐7 CPLX and separated BMP‐7 PD were subjected to cleavage by indicated MMPs. Samples were loaded onto a 10%‐20% SDS‐PAGE gradient gel, followed by western blot analysis of cleavage products employing antibodies (mab2/mab33, 1:1 mixture) against BMP‐7 PD

Previously, it was shown that active TGF‐β is able to stimulate matrix metalloproteinase (MMP) expression.^55^ Furthermore, MMP cleavage of the TGF‐β PD LAP was proposed as mechanism to release TGF‐β GF from the ECM.^31^, ^32^, ^33^ To evaluate the existence of a potential similar MMP‐driven ECM activation mechanism for BMPs, the hypothesis was tested whether the BMP‐7 PD also serves as a substrate for MMPs. For this purpose, an in vitro cleavage screening assay was undertaken testing different recombinantly expressed and purified representatives of MMP family subgroups (Figure 2A) and BMP‐7 PD and BMP‐7 CPLX as substrates. In our screen, MMP‐2 and –9 represented the gelatinases, MMP‐3 the stromelysins, MMP‐7 the matrysins, MMP‐8 and ‐13 the collagenases, and MMP‐12 the elastases.^56^ Prior to BMP‐7 PD incubation, MMP activity was assessed through fluorescence increase after incubation with a specific MMP fluorogenic substrate. Cleavage of the substrate resulted in a fluorescence signal at 500 nm (Figure 2B). All tested MMPs were able to cleave the BMP‐7 PD, either alone, or when complexed to its cognate GF (Figure 2C). Thereby, we observed varying BMP‐7 PD cleavage efficiencies among MMPs, since MMP‐7, and ‐9 only showed moderate activity under the chosen conditions. Interestingly, a considerable number of MMP‐mediated cleavage events yielded in BMP‐7 PD fragments of similar sizes approximately at 30, 25, and 20 kDa (Figure 2C).

### 3.3 Mapping of MMP cleavage site within the BMP‐7 PD

**FIGURE 3:**
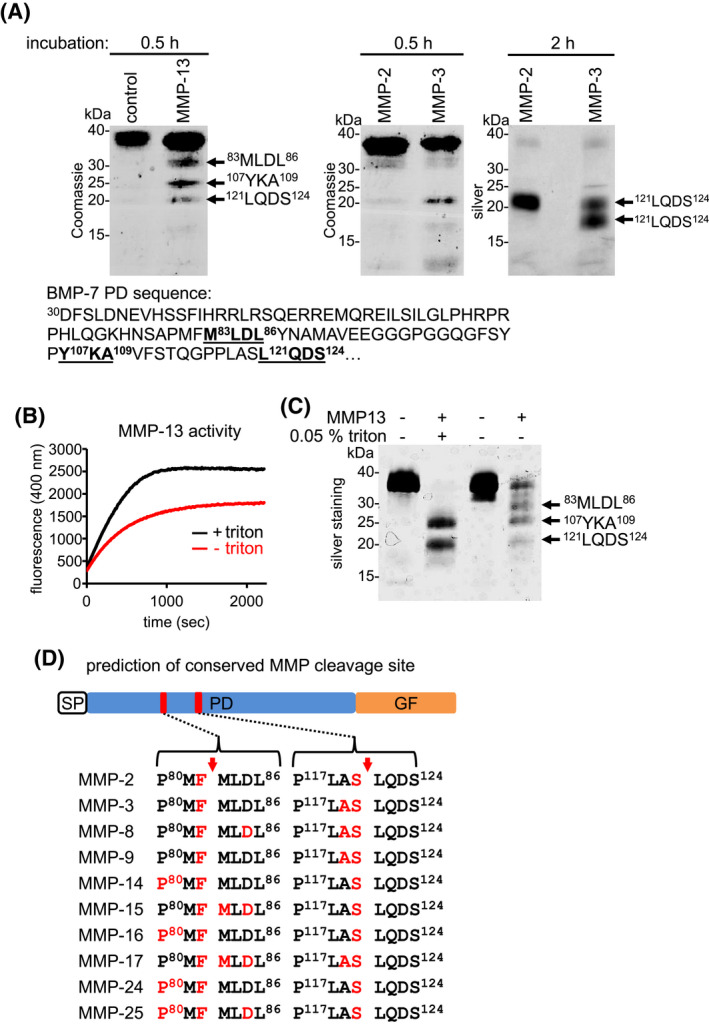
Identification of MMP cleavage site within BMP‐7 PD. A, (top) N‐terminal Edman sequencing of BMP‐7 PD fragments generated by MMP‐2, MMP‐3, and MMP‐13 cleavage after indicated incubation times. Resulting BMP‐7 PD fragments were separated by SDS‐PAGE in a 10%‐20% gradient gel followed by Coomassie staining. (bottom) N‐terminal amino acid sequences of MMP‐13‐mediated BMP‐7 PD fragments highlighted in black bold and underlined letters. B, MMP‐13 activity in the presence or absence of 0.05% Triton X‐100 was monitored using a specific substrate. C, BMP‐7 PD cleavage (incubation time: 2 h) by MMP‐13 in presence or absence of Triton X‐100 followed by 10%‐20% gradient SDS‐PAGE and silver staining. D, MMP cleavage site prediction within BMP‐7 PD using the CleavPredict bioinformatics platform. All evaluated MMPs were predicted to cleave BMP‐7 PD at the same positions identified by Edman degradation after MMP‐13 cleavage. Predicted amino acids located in position 1 (P1) (position right before the cleavage site) are highlighted in red. Arrows indicate position of predicted cleavage sites

To identify specific MMP cleavage sites within the BMP‐7 PD, N‐terminal Edman sequencing of cleavage products was performed after incubation with MMP‐2, MMP‐3, and MMP‐13. Using this method, the N‐terminal sequence of a consensus cleavage product at a size of 20 kDa for MMP‐2, MMP‐3, and MMP‐13 was identified starting with ^121^LQDS^124^. In addition, the N‐terminal amino acid sequence of two other peptides could be identified after cleavage by MMP‐13: ^83^MLDL^86^, and ^107^YKA^109^ (Figure 3A). At 0.5 hours incubation time, a higher cleavage efficiency of MMP‐13 was observed in comparison to MMP‐2 and ‐3 (Figure 3A). After 0.5 hours incubation in presence of MMP‐2 and ‐3, minor amounts of the first cleavage products could be also detected at 30 kDa similar to the fragment starting with ^83^MLDL^86^ identified after MMP‐13 cleavage. After 2 hours of incubation, quantitative MMP‐2 and ‐3 cleavage was observed yielding the ^121^LQDS^124^ fragment at 20 kDa as well as an additional fragment at about 17 kDa with the same N‐terminal ^121^LQDS^124^ sequence most probably generated by a secondary cleavage event further downstream towards the C‐terminus (Figure 3A).

Our assays revealed that MMP‐13 cleavage activity was most effective in the presence of 0.05% Triton X‐100 (Figure 3B). Therefore, the MMP‐13‐induced cleavage pattern of BMP‐7 PD in the presence or absence of triton was assessed. In the presence of triton, full length BMP‐7 PD, as well as the fragment starting with ^83^MLDL^86^ were not detectable, leading to a more prominent presence of fragments starting with ^121^LQDS^124^ and ^107^YKA^109^ (Figure 3C). This finding suggests that fragments starting with ^121^LQDS^124^ and ^107^YKA^109^ are products of a secondary cleavage event derived from the fragment starting with ^83^MLDL^86^ produced in the primary cleavage event.

Overall, our data led us to the hypothesis that MMP‐2, MMP‐3, and the other tested MMPs initially process BMP‐7 PD at the same cleavage sites as MMP‐13. However, the cleavage products may be further processed by different secondary cleavage events. To further explore the possibility of a general MMP cleavage site within BMP‐7 PD, the publicly available MMP cleavage prediction platform, CleavPredict,^57^ was utilized. With the help of this software which is based on *P*roteomic *I*dentification of *P*rotease *C*leavage *S*ites (PICS) using human peptide libraries,^57^ potential cleavage sites within the BMP‐7 PD for 11 representative MMPs could be predicted. Interestingly, all evaluated MMPs were predicted to cleave BMP‐7 PD within the same sites identified by Edman degradation after MMP‐13 incubation (Figure 3D).

### 3.4 MMPs specifically cleave PDs of TGF‐β superfamily members

**FIGURE 4:**
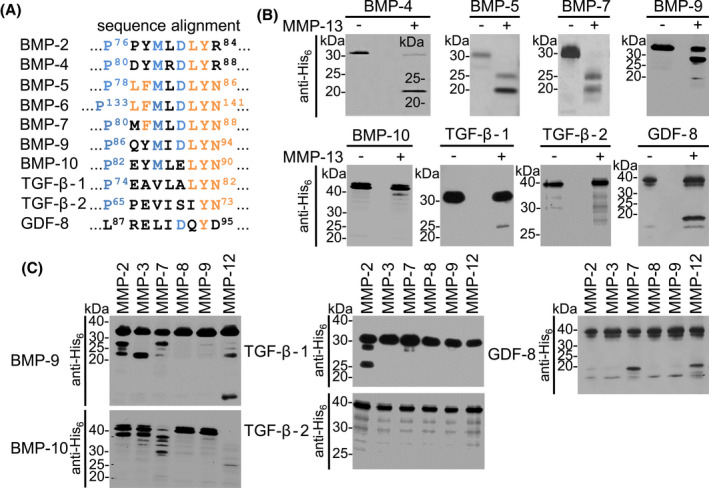
Identified MMP cleavage site is conserved among TGF‐β superfamily members. A, Sequence alignment of PD sequences of BMP‐2, ‐4, ‐5, ‐6, ‐7, ‐9, ‐10, GDF‐8, TGF‐β‐1, and ‐2 by Clustal Omega showed that the identified MMP cleavage site is partially conserved with the exception of GDF‐8. Conserved amino acid residues among evaluated TGF‐β superfamily members are highlighted in blue. B, Proteolytic processing of TGF‐β superfamily PDs by MMP‐13 assessed by western blotting using anti‐His_6_ antibody. C, TGF‐β superfamily PDs subjected to proteolytic cleavage by representative members of the MMP family. Cleavage samples were analyzed by 10%‐20% SDS‐PAGE followed by western blotting using anti‐His_6_antibody

**FIGURE 5:**
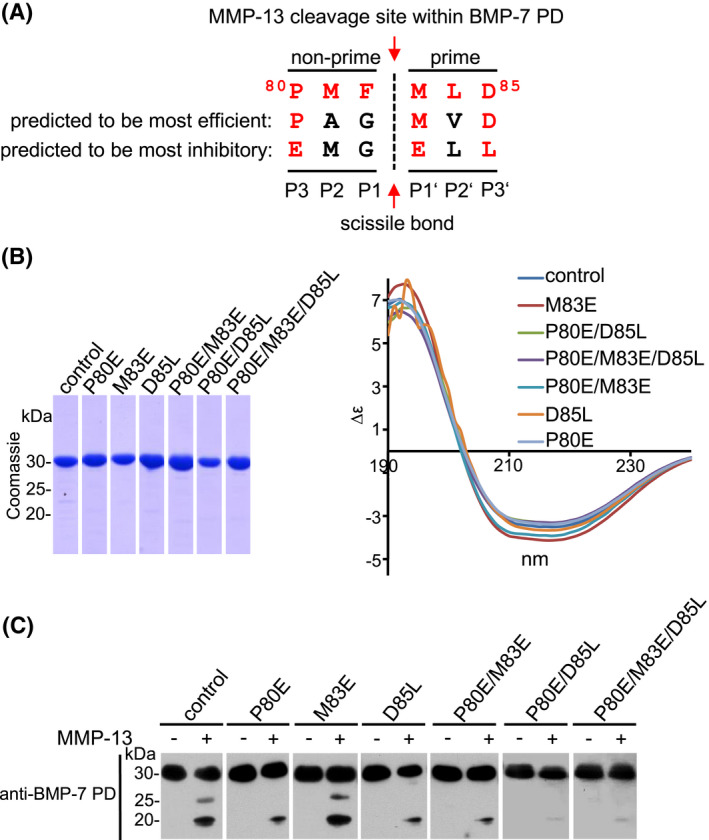
Characterization of MMP‐13 cleavage site within BMP‐7 PD. A, Introduced point mutations within identified MMP‐13 cleavage site in BMP‐7 PD. Identified MMP‐13 cleavage motif within BMP‐7 PD is indicated in red. The most efficiently cleaved motif and residues to be most inhibitory for MMP‐13 cleavage were previously predicted.^58^ B, Evaluation of integrity, purity, and secondary structure of mutant BMP‐7 PD variants by SDS‐PAGE (12.5% gel stained with Coomassie), and circular dichroism. C, BMP‐7 PD variants carrying point mutations: P80E, M83E, and D85L, double point mutations: P80E/M83E and P80E/D85L, as well as a triple point mutation: P80E/M83E/D85L were subjected to MMP‐13 cleavage. Resulting fragments were separated by 10%‐20% gradient SDS‐PAGE and visualized by western blotting using anti‐BMP‐7 PD antibody (mab2/mab33, 1:1 mixture)

Sequence alignment of PDs showed that the identified MMP cleavage site ^80^PMFMLD^85^ within the BMP‐7 PD is partially conserved among other members of the TGF‐β superfamily, except for GDF‐8 (Figure 4A). Subjecting PD sequences of BMP subgroup representatives to in silico cleavage by CleavPredict revealed that most MMPs would also utilize this site (Figure S2). Previous studies had shown that Pro in P3 in the non‐prime region before the scissile bond of the cleavage site (Figure 5A) is considered the most important amino acid required for MMP‐13 recognition, followed by Leu or Met in position P1′, and acidic residues in P3′ of the prime region.^58^ The alignment showed that the corresponding residues identified in BMP‐7 PD P^80^, M^83^, and D^85^ were conserved among BMP family members (Figure 4A). In addition, we found the motif ^86^LYN^88^ to be conserved for most PDs in positions P4′‐P6′ after the scissile bond.

To experimentally validate that PDs of TGF‐β family members serve as substrates for MMP‐13 and other MMPs, an in vitro cleavage screen with representatives of TGF‐β and BMP subgroups was performed. Under the chosen conditions, we observed that MMP‐13 was able to process all tested PDs with varying efficiencies (Figure 4B). As expected, MMP‐13 cleavage of the PD of BMP‐5, which belongs to the BMP‐5, ‐6, ‐7 subgroup, yielded the same cleavage pattern as seen for the BMP‐7 PD (Figure 4B). After cleavage of the BMP‐4 PD, a representative of the BMP‐2, ‐4 subgroup, only the 20 kDa fragment could be detected (Figure 4B). A cleavage screen of MMP subgroup representatives with PDs of BMP‐9 and ‐10 which both constitute their own BMP subgroup, revealed differences and similarities in resulting fragment patterns and cleavage efficiencies (Figure 4C). Similar cleavage results were observed after MMP‐2, ‐7, and ‐13 incubation (Figure 4B,C), while BMP‐10 PD processing by MMP‐12 was more effective than that of BMP‐9 PD. Interestingly, MMP‐8 and ‐9 did not process PDs of this subgroup.

TGF‐β‐1 PD was efficiently cleaved by MMP‐2 (Figure 4C) as already described.^32^, ^59^ Also upon MMP‐13 cleavage, a fragment at around 25 kDa could be observed in minor amounts (Figure 4B). In contrast, the TGF‐β‐2 PD was not susceptible to cleavage by the tested MMPs and only a minor degradation was found upon incubation with MMP‐2, ‐12, and ‐13 (Figure 4B,C).

As GDF‐8 lacks most of the residues of the identified, conserved MMP cleavage site, ^80^PMFMLD^85^ (Figure 4A), we expected that GDF‐8 might be resistant against MMP cleavage. However, in presence of MMP‐7, ‐12 and ‐13, a cleavage product was detected around 19 kDa (Figure 4B,C).

### 3.5 The ^80^PMFMLD^85^ motif is crucial for efficient MMP‐13 cleavage of BMP‐7 PD

To validate the relevance of the identified ^80^PMFMLD^85^ motif we attempted to inactivate MMP‐13 processing of BMP‐7 PD by introducing point mutations at critical positions (Figure 5A). Thereby, amino acid substitutions were guided by previous predictions.^58^ BMP‐7 PD mutants were overexpressed in *E coli* and affinity purified to more than 95% purity as assessed by SDS‐PAGE and Coomassie staining (Figure 5B). The generated BMP‐7 PD mutant variants included the single point mutations P80E, M83E, and D85L, the double point mutations P80E/M83E, and P79E/D84L, as well as the triple point mutation P80E/M83E/D85L. All chosen point mutations did not result in secondary structure changes as assessed by circular dichroism (CD) spectroscopy (Figure 5B). All mutated BMP‐7 PD variants were subjected to MMP‐13 cleavage and the resulting fragments were analyzed via western blot analysis (Figure 5). In all mutant variants apart from M83E, production of the 25 kDa fragment was abolished and the presence of the 20 kDa fragment was significantly reduced (Figure 5C).

### 3.6 Localization of the MMP‐13 cleavage site within a three‐dimensional structure model of BMP‐7 CPLX

**FIGURE 6:**
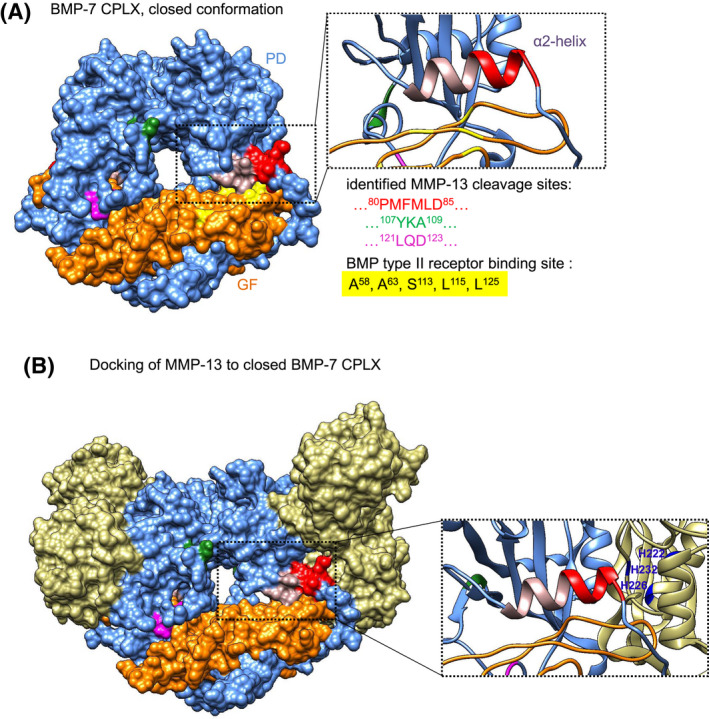
Model of MMP‐13 cleaving within the inhibitory α2‐helix of the PD in the closed‐ring BMP‐7 CPLX conformation. A, Localization of the MMP‐13 cleavage sites within a three‐dimensional structure model of BMP‐7 CPLX (surface representation). BMP‐7 CPLX is presented in the closed‐ring shape conformation, and identified cleavage regions are highlighted: ^80^PMFMLD^85^ (red), ^107^YKA^109^ (green), ^121^LQD^123^ (magenta). Blue: PD residues, orange: GF residues, inhibitory α2‐helix: light purple, yellow: crucial GF residues interacting with the BMP type II receptors. Area outlined by dashed box is also shown as twofold magnification in ribbon representation. B, Docking of two MMP‐13 molecules (beige) to BMP‐7 CPLX in closed ring‐shape conformation. A twofold magnification of area outlined by dashed box in ribbon representation shows residues of the MMP‐13 catalytic site (H^222^, H^226^, H^232^ marked in blue) in proximity to the ^80^PMFMLD^85^ cleavage site (marked in red)

Binding of BMP‐7 CPLX to fibrillin‐1 induces a conformational change of the entire CPLX from an open bioactive V‐shape to a closed latent ring‐shape.^42^ This inactivation of BMP GF occurs due to a structural re‐arrangement of the two PDs leading to blockage of the BMP type II receptor‐binding site on the GF by the α2 helix of the PD^42^ (Figure 6A). By localizing the scissile bond of the identified MMP‐13 cleavage site (between F^82^ and M^83^) within the PD in a three‐dimensional closed ring‐shape structure model of BMP‐7 CPLX (Figure 6A), we found that it resides within the α2 helix. Since molecular docking of MMP‐13 and the closed BMP‐7 CPLX suggested that this site was accessible (Figure 6B), we hypothesized that MMP‐13‐mediated cleavage of latent fibrillin‐1‐bound BMP‐7 CPLX leads to removal of the α2‐helix resulting in release of bioactive BMP‐7 GF.

### 3.7 MMP‐13 cleavage leads to release of active BMP‐7 GF from ECM‐bound BMP‐7 CPLX pools

**FIGURE 7:**
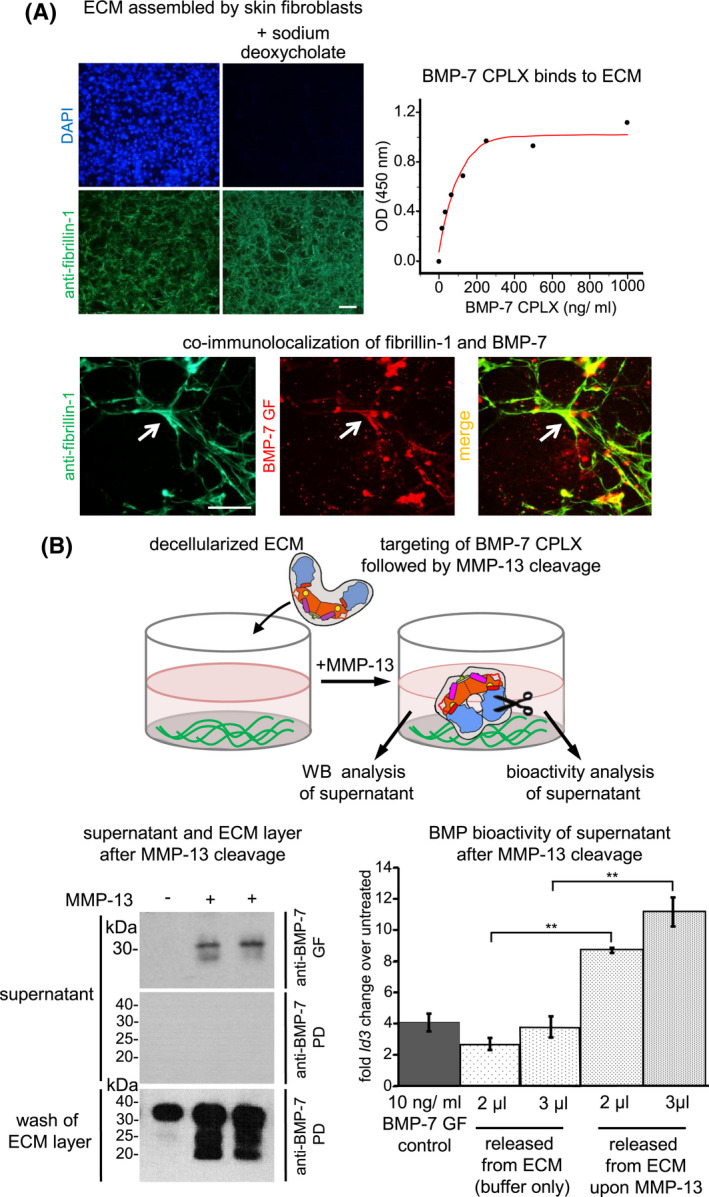
MMP‐13 cleavage of the BMP‐7 PD leads to release of bioactive GF from ECM‐targeted BMP‐7 CPLX. A, Targeting of BMP‐7 CPLX to ECM fibers assembled by skin fibroblasts. Top left, Immunofluorescence of assembled ECM fibers by primary mouse skin fibroblasts stained with anti‐fibrillin‐1 antibody and DAPI before and after decellularization with deoxycholate. Scale bar: 100 µm. Top right, ELISA‐style interaction assay of recombinant BMP‐7 CPLX with the decellularized ECM layer. Anti‐BMP‐7 GF antibody was used for detection. Bottom, Co‐immunofluorescence analysis using anti‐fibrillin‐1 and anti‐BMP‐7 GF antibodies after incubation of recombinant BMP‐7 CPLX with decellularized ECM fibers. Arrows point to yellow signals indicating co‐localization between fibrillin‐1 and BMP‐7. Scale bar: 50 µm. B top, Scheme illustrating the design of MMP‐13‐mediated BMP‐7 GF release experiment from ECM‐targeted fraction. Bottom left, Supernatant and ECM layer after MMP‐13 incubation were analyzed by western blot for BMP‐7 presence. Bottom right, Stimulation of C2C12 cells with supernatant after MMP‐13 incubation of BMP‐7 CPLX bound to decellularized ECM *Id3* transcript levels were used as read‐out for BMP‐7 bioactivity. Error bars show standard deviation of three independent experiments (N = 3). Statistical analysis was by an unpaired two‐tailed Student's t test. **P ≤ .01

To test whether MMP‐13 is able to cleave and activate BMP‐7 CPLX from ECM‐bound pools, BMP‐7 CPLX was targeted to fibrillin fibers assembled by primary fibroblasts (Figure 7A). Potential release of bioactive GF after MMP‐13 cleavage into the supernatant was monitored via western blotting and BMP bioactivity assays. First, efficient binding of BMP‐7 CPLX to decellularized fibrillin‐1 fibers was demonstrated by detecting co‐localizing immunofluorescence signals as well as by ELISA showing a concentration‐dependent signal increase (Figure 7A). In a subsequent step, wells with fibrillin fibers decorated with BMP‐7 CPLX were incubated with MMP‐13. Western blot analysis showed the presence of released BMP‐7 GF into supernatant only when MMP‐13 was added (Figure 7B). PD fragments could be only detected in the washed ECM layer, suggesting that upon cleavage they remain attached to the ECM. To assess whether released BMP‐7 GF was bioactive, the supernatant was added to C2C12 cells and the mRNA expression of the endogenous BMP response gene *Id3* was measured (Figure 7B). Upon addition of MMP‐13, an approximate fourfold increase in BMP activity was detected indicating that the BMP‐7 GF was released in bioactive form (Figure 7B).

### 3.8 MMP‐13 cleavage of BMP‐7 PD results in conformational change and CPLX disintegration

**FIGURE 8:**
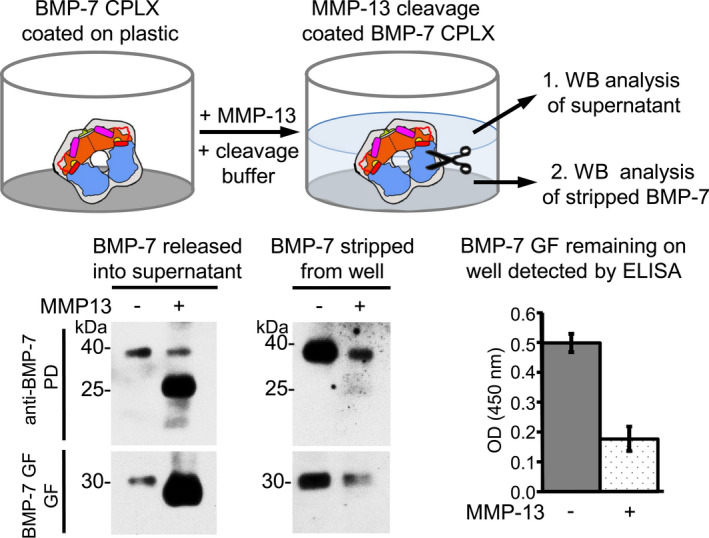
MMP‐13 cleavage of BMP‐7 PD on solid phase leads to BMP‐7 CPLX disintegration and GF release. Top, Scheme to illustrate the set‐up of the conducted experiment: BMP‐7 CPLX was coated to microtiter plate wells and subsequently incubated with MMP‐13 in cleavage buffer. The aspirated supernatant after MMP‐13 cleavage was TCA precipitated and subjected to 10%‐20% SDS‐PAGE and western blot analysis using antibodies against BMP‐7 PD and BMP‐7 GF for detection. In addition, amounts of BMP‐7 CPLX remaining on the well surface were stripped (300 mM NaCl and 200 mM acetic acid), TCA precipitated and subjected to western blot analysis. Bottom left, Western blot analysis of BMP‐7 PD and GF in supernatant and in fraction stripped from well after MMP‐13 incubation in cleavage buffer. Bottom right, Determination of remaining amounts of coated BMP‐7 GF on well after MMP‐13 incubation by direct ELISA using anti‐BMP‐7 GF antibody

To investigate whether MMP‐13 cleavage also leads to GF release when the CPLX is immobilized via hydrophobic residues to a non‐ECM solid phase, we performed cleavage studies with plastic‐coated CPLX pools. For this purpose, BMP‐7 CPLX was coated onto wells of microtiter plates, and was subsequently incubated with MMP‐13 for 2 hours at RT in TC buffer (Figure 8). After MMP‐13 cleavage, the supernatant was collected and subjected to SDS‐PAGE and western blot analysis (Figure 8). In addition, BMP‐7 CPLX remaining on the plate was stripped (in 300 mM NaCl, 200 mM acetic acid) and analyzed by western blotting. Similar to the release experiment from fibrillin‐1 fibers, it was found that upon MMP‐13 cleavage BMP‐7 GF was released into the supernatant. However, in contrast to the ECM release experiment (Figure 7B), BMP‐7 PD fragments were simultaneously released into the supernatant. Efficient release of BMP‐7 GF could be also confirmed by ELISA detecting less than 40% of BMP‐7 GF still immobilized to the plate (Figure 8).

**FIGURE 9:**
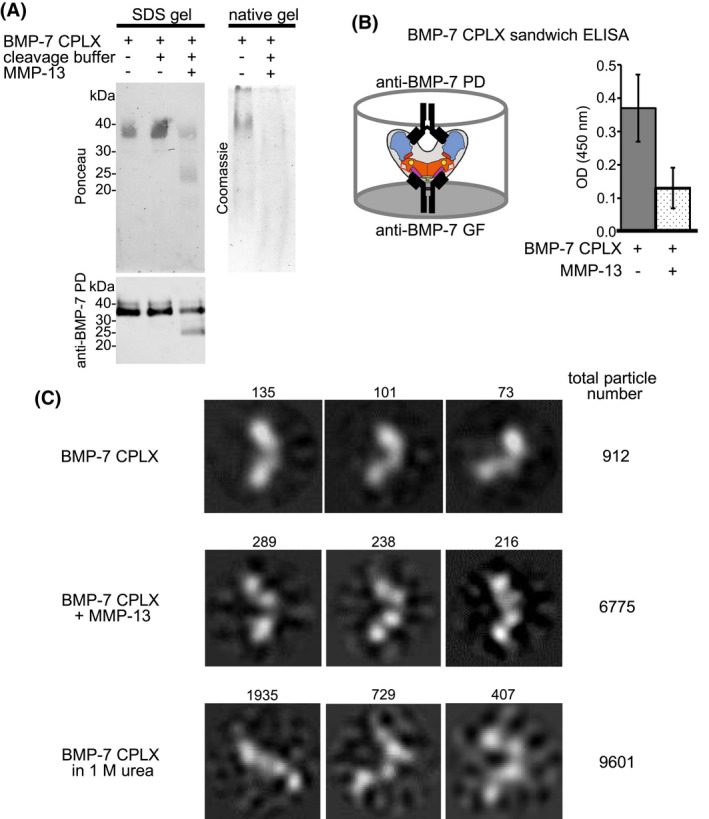
BMP‐7 PD cleavage by MMP‐13 leads to conformational change of BMP‐7 CPLX. A, SDS‐PAGE, native‐PAGE, as well as western blot (mab33) analysis of BMP‐7 PD after MMP‐13 cleavage. B left, Illustration of sandwich ELISA set‐up: anti‐BMP‐7 GF antibody was immobilized, and anti‐BMP‐7 PD antibody (mab33) was used for detection. Right, Remaining intact BMP‐7 CPLX after MMP‐13 incubation was detected by sandwich ELISA. C, Single particle TEM analysis of BMP‐7 CPLX after MMP‐13 incubation (same samples as shown in [A]), as well as after dialysis into 1 M urea. Depicted images represent class averages of multiple micrographs taken from individual molecules. Number of averaged individual molecules is given above each image (box size = 29.4 × 29.4 nm)

To gain further insight into how BMP‐7 PD cleavage by MMP‐13 affects BMP‐7 CPLX stability, we analyzed samples after in solution cleavage by SDS‐PAGE, native‐PAGE, sandwich ELISA, and single particle TEM. Interestingly, at 50% BMP‐7 PD cleavage, as assessed by Ponceau and western blot analysis, the CPLX signal was not detectable by Coomassie staining on native gels (Figure 9A). To exclude the possibility that the loss of a distinct Coomassie band on native gels was not caused by major aggregation of the BMP‐7 CPLX after PD cleavage, we performed dynamic light scattering (DLS) analysis before and after MMP‐13 cleavage (Figure S3). Our analysis showed that MMP‐13 cleavage did not lead to a decrease of the peak representing monomeric CPLX molecules at a particle size of 10 nm (Figure S3). The peak representing aggregated particles of an average size of 100 nm showed even a slight decrease suggesting that MMP‐13‐mediated cleavage of the PD led to a decrease rather than an increase of aggregation (Figure S3). To assess CPLX stability after MMP‐13 cleavage we performed sandwich ELISA. However, when BMP‐7 CPLX after in solution cleavage was transferred to wells pre‐coated with anti‐BMP‐7 GF antibody, followed by incubation with anti‐BMP‐7 PD detection antibody, still 30% of signal could be detected compared to the non‐cleaved control (Figure 9B).

Single particle TEM analysis of MMP‐13‐cleaved BMP‐7 CPLX samples shown in Figure 9A revealed a conformational change of processed CPLX particles. Representative class averages of EM micrographs of the most frequent particles revealed that BMP‐7 CPLX cleaved by MMP‐13 adopts a conformational change characterized by a widened angle between the PD arms and a four‐subparticle appearance. This appearance was similar to the conformational change induced by the addition of 1 M urea which results in partial CPLX unfolding leading to partial PD displacement from the GF.^42^

These findings suggested the possibility that processing of one PD per CPLX molecule leads to a conformational change in each molecule and, therefore, to an unfocused migration in native gels. The sandwich ELISA shows that less than 30% CPLX seems to be stable enough for detection. However, the unfolding leads to an unfocused migration in native‐PAGE and, therefore, lack of staining intensity at the expected position.

### 3.9 Molecular docking suggests that BMP‐7 CPLX cleavage by MMP‐13 requires PD displacement

**FIGURE 10:**
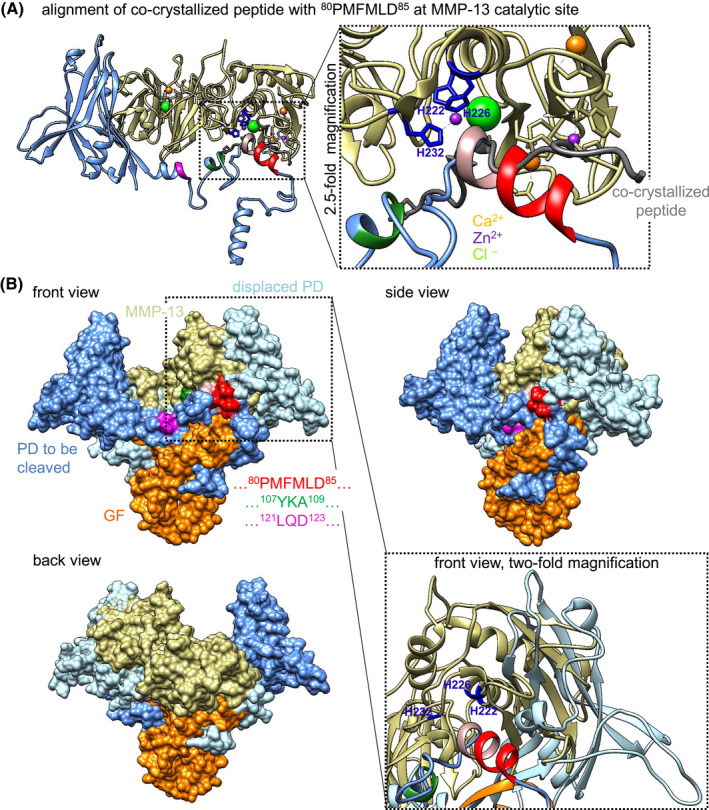
MMP‐13 co‐localizes with one BMP‐7 PD to accomplish cleavage of the other PD in the open CPLX conformation. A, Docking of one BMP‐7 PD in open conformation to the MMP‐13 catalytic site. Residues of the catalytic site: H^222^, H^226^, H^232^ (all in blue), as well as cleavage regions ^80^PMFMLD^85^ (red), ^107^YKA^109^ (green), and ^121^LQD^123^ (magenta) are marked. The magnified dashed box shows co‐localization of the α2‐helix (light purple) carrying the cleavage site ^80^PMFMLD^85^ with the co‐crystallized peptide (gray) of the MMP‐13 crystal structure serving as positive control. B, Surface models of MMP‐13 (beige) and BMP‐7 CPLX in open V‐shape conformation suggesting MMP‐13 needs to bend one PD (light blue) to cleave the other (blue). Magnified (twofold) dashed box shows co‐localization of MMP‐13 and BMP‐7 PD in ribbon representation. GF residues: orange

To understand how MMP‐13 processing of BMP‐7 CPLX may occur on a molecular level in silico docking experiments were conducted. The center of the MMP‐13 catalytic site is composed of H^222^, H^226^, and H^232^ that capture divalent metal ions with their aromatic rings to polarize water molecules that subsequently attack peptide bonds to perform the proteolysis (Figure 10A). Our docking results revealed that positioning of the ^83^MLDL^86^ stretch of one PD at the MMP‐13 active cleft allows for further processing of the BMP‐7 PD at the subsequent cleavage sites ^107^YKA^109^ and ^121^LQD^123^ due to the interspacing loop regions that allow for flexibility of the PD structure at these sites during the cleavage. To validate the imaging approach, MMP‐13 of our in silico cleavage model was aligned to MMP‐13 structure co‐crystallized with an N‐terminal fragment of its activation peptide in its active cleft. Eight such co‐crystallized peptides, originating from the MMP‐13 activation peptide after cleavage, consisted of an α‐helix or random coil.^60^ This agrees with our experimental data suggesting that the three MMP‐13 cleavage sites of BMP‐7 PD reside within the second and fourth α‐helices at the N‐terminal region of the PD (Figure 10A). The ^83^MLDL^86^ stretch aligns perfectly with the co‐crystallized peptide used in this in silico experiment (Figure 10A).

Next, we aligned our generated MMP‐13/BMP‐7 PD cleavage model (Figure 10A) to PDs of our BMP‐7 CPLX model in open V‐shape conformation. Thereby, we found that in this open CPLX conformation MMP‐13 would need to bend or even displace one PD to gain access for efficient processing of the other PD (Figure 10B).

## 4 DISCUSSION

Although many studies addressed the mechanisms of TGF‐β activation,^29^, ^30^, ^32^, ^34^, ^59^, ^61^, ^62^ the required pathways for cellular utilization of other TGF‐β superfamily members such as BMPs remain largely unknown. Since the discovery of BMPs as pluripotent cytokines extractable from bone matrix, it has been speculated how BMPs targeted to the ECM become released and activated.

In this study, we uncovered a new proteolytic PD cleavage mechanism involving MMPs to release BMP GFs from FMF‐stored ECM pools. In this context, BMP PDs not only mediate efficient targeting and sequestration of BMP GFs upon ECM binding, but also allow controlled release of bioactive GF upon specific cleavage by MMPs. Our previous studies showed that binding of BMP‐7 CPLX to fibrillin‐1 induces a conformational change of the entire CPLX from an open bioactive V‐shape to a closed latent ring‐shape (Figure 6A). In this closed conformation, a structural re‐arrangement of the two PDs leads to blockage of the BMP type II receptor‐binding sites on the GF by the α2‐helix of the PD (Figure 6).^42^ Here, in our proposed MMP‐13‐mediated BMP‐7 activation mechanism, recognition of the ^80^PMFMLD^85^ motif followed by PD processing of the scissile bond between F^82^ and M^83^ leads to unfolding and disintegration of the entire CPLX resulting in release of the active GF dimer.

Our investigations revealed interesting molecular aspects of this BMP activation mechanism. Molecular docking experiments (Figure 6B) suggested the potential necessity of two MMP‐13 monomers for efficient PD processing in the closed BMP‐7 CPLX ring‐shape conformation, to effectively release the active GF dimer. However, in the open V‐shape CPLX, efficient scissile bond cleavage by MMP‐13 at the ^80^PMFMLD^85^ site in one PD requires partial displacement of the other PD, suggesting a 1:1 stoichiometry (Figure 10).

Our in solution data suggest that cleavage of potentially only one PD is sufficient to result in unfolding and disintegration of the entire CPLX (Figure 6A). A possible explanation for this observation is that PD self‐interaction contributes significantly to CPLX stability.^42^ Previously, we had also shown that BMP‐7 PDs compete with BMP type II receptors for the same binding site on the GF, and that upon type II receptor binding, both PDs are displaced as a dimer.^39^ PD dimers could be also detected in solution when the CPLX was step by step separated into its components by dialysis into increasing concentrations of urea.^42^ This suggests that PD dimers serve as a molecular clamp only exerting tight complexation of the GF dimer, when the dimer is intact. Our previous in solution data also showed that CPLX unfolding and disintegration takes place upon dialysis into increasing concentrations of urea (1‐4 M).^42^ Here, our EM data showed partial unfolding of the CPLX already at a concentration of 1 M urea (Figure 9C), which we previously demonstrated is accompanied by PD displacement as dimer.^42^ This poses the possibility that the analyzed molecules with widened angle and four‐subparticle appearance represent GF dimers from which PD dimers are about to be displaced in the 1 M urea sample. The similar appearance between BMP‐7 CPLX molecules after dialysis into 1 M urea and MMP‐13 cleavage further support our notion that PD cleavage leads to CPLX unfolding and release of the PD dimer clamp.

In the BMP PD cleavage assays conducted, we found that presence of 0.05% Triton X‐100 served as an optimal concentration for efficient MMP‐13 cleavage (Figure 3B,C). It is known that MMP activity is dependent on the detergent concentration used. To avoid adverse effects, it is crucial to determine the optimal detergent concentration. At low concentrations, detergents act as monomers that may stabilize and activate MMPs. However, at high concentrations they form micelles that might sequester and inhibit MMP activity.^35^, ^63^ In absence of Triton, BMP‐7 PD processing by MMP‐13 occurred with a reduced turn‐over rate yielding three fragments starting with ^83^MLDL^86^, ^121^LQDS^124^, and ^107^YKA^109^ (Figure 3C). However, in presence of Triton the ^83^MLDL^86^ fragment disappeared leading to an increased presence of the other two fragments (Figure 3B). This finding suggests that the fragment starting with ^83^MLDL^86^ is produced during the primary cleavage event and ^121^LQDS^124^ and ^107^YKA^109^ are products of subsequent cleavage events. These experimental data are also in line with our in silico docking results which showed that the F^82^‐M^83^ scissile bond was most surface accessible in comparison to P^106^‐Y^107^, or S^120^‐L^121^ in the closed ring‐shape BMP‐7 CPLX conformation. Our experimental data also suggest that the S^120^‐L^121^ scissile bond is less accessible in absence of Triton in solid phase cleavage assays indicated by a less pronounced presence of the PD cleavage product at 20 kDa (Figures 8 and 9).

Using CleavPredict,^58^ a cleavage site composed of six amino acid residues was predicted, three non‐prime (amino acids downstream to the cleaved scissile bond) and three prime (amino acids upstream to the cleaved scissile bond) (Figure 5A). For most efficient MMP‐13 cleavage, a general cleavage site was proposed based on a pronounced presence of a rigid Pro in P3 (non‐prime), and Leu, Ile, or Met in P1′ (prime), followed by small or acidic residues in P3′ in the identified substrates.^58^ In all mutated BMP‐7 PD variants, apart from M83E, production of the fragment starting with ^83^MLDL^86^ was abolished and the fragments starting with ^121^LQDS^124^ and ^107^YKA^109^ were significantly reduced (Figure 5C). This result is supportive of the hypothesis that P^80^ and D^85^are required for PD cleavage by MMP‐13 but disagrees with the assumption that M^83^ is crucial for MMP‐13 processing, since mutation of this residue did not affect the cleavage pattern (Figure 5C).

Our sequence alignment analysis showed that the identified ^80^PMFMLD^85^ motif in the BMP‐7 PD was conserved among other BMP PDs (Figure 4A, Figure S2), suggesting a general MMP‐mediated BMP PD cleavage mechanism. Our BMP PD cleavage screen revealed similarities but also differences to BMP‐7 PD processing. BMP‐5 and ‐7 belong to the same BMP subgroup and showed an identical cleavage pattern for MMP‐13 (Figure 4B). However, BMP‐4 PD processing did not yield the band at 25 kDa suggesting more efficient secondary cleavage. This may be due to different amino acid residues present in the P1 and P2 positions of the non‐prime region of the cleavage site. Also, in BMP‐9 and 10 PDs the most significant changes of the cleavage motif are within this region resulting in less efficient processing by MMP‐13. More specifically, BMP‐10 PD lacks an Asp (D) in the P3′ position of the prime region that is predicted to be required for efficient cleavage (Figure 5A).^58^ This may explain the almost diminished MMP‐13 cleavage of BMP‐10 PD compared to BMP‐9 PD (Figure 4B). However, our results also showed that most likely these changes protect BMP‐9 and ‐10 PD from MMP‐8 and ‐9 cleavage but make them more susceptible to MMP‐12 processing in comparison to BMP‐7 (Figure 4C). It is also conceivable that the different conformations of the BMP PDs impact MMP cleavage efficiency in the conducted assays. Based on the sequence homology, it can be assumed that BMP‐9 and ‐10 PDs fold similarly but differently than BMP‐7 PD.

In TGF‐β‐1 and ‐2 most critical residues of the identified MMP motif in BMP‐7 PD are altered. However, maintaining a Pro in P3 and some conserved residues in the P4′‐P6′ prime region most probably allows MMP‐13 processing of both TGF PDs to a minor extent. Interestingly, we observed only very limited MMP‐2 cleavage of TGF‐β‐2 PD in comparison to TGF‐β‐1 (Figure 4C). This suggests that the presence of a second proline at the P2 position of the non‐prime region is inhibitory for efficient MMP processing (Figure 4A). As GDF‐8 lacks most residues predicted to be critical for MMP recognition and processing, we expected it to be inert against MMP cleavage. However, we observed that after MMP‐7, ‐12, and ‐13 incubation, a cleavage product at around 19 kDa was detected. According to our predictions using CleavPredict, substitution of Pro to Leu in P1 which is found in the GDF‐8 PD sequence, is not sufficient to block MMP‐13 cleavage. Based on the alignment shown in Figure 4A, the GDF‐8 PD sequence still contains a conserved Asp (D) residue in P3′ of the ^80^PMFMLD^85^ motif. Also, Leu in P1′ and Ile in P2′ were found suitable for MMP cleavage using trypsin‐generated human peptide libraries.^58^ Furthermore, a degradation product of about 19 kDa is also obtained upon cleavage of GDF‐8 PD by TLL2. The TLL2 cleavage site ^99^DDSSDGSLE^107^ was identified to be located three amino acids further downstream to the identified MMP cleavage site.^37^ Based on the similar sizes, we can speculate that this region may be also prone to proteolytic degradation by MMPs. Since TLL2 cleavage at this site showed to weaken critical PD‐GF interactions leading to activation of the GF from its latent CPLX form,^37^ the newly found proteolytic susceptibility of GDF‐8 PD opens up further investigation to study similar activation mechanisms mediated by MMPs.

The investigated MMP‐mediated cleavage events yielded in BMP‐7 PD fragments of similar sizes approximately at 30, 25, and 20 kDa (Figure 2C) which remain attached to the ECM layer (Figure 7B). Previously, we reported that the BMP‐7 CPLX binds to fibrillin‐1 via a specific epitope residing within Gly^74^ ‐ Arg^184^ of the PD. Therefore, it is possible that all three PD cleavage products (starting with M^83^, Y^107^, or L^121^) generated by MMP‐13 cleavage still contain the high affinity binding site for fibrillin‐1 and are, therefore, not released from FMF under the chosen buffer conditions. This may suggest that PD fragments remaining on the FMF may block targeting of freshly produced BMP CPLXs. However, it is certainly possible that further degradation reactions occur in vivo to finally clear the remaining PD fragments. The fate of PD fragments has to be viewed in a context‐dependent manner. In situations with little ECM present such as during embryogenesis, PD fragments may be released freely to exert their own functional role. It was already reported that certain BMP‐7 PD fragments called bone forming peptide 1 and 2 (BFP‐1 and ‐2) can induce osteogenesis more potently than BMP‐7 GF in in vitro and in vivo experiments.^64^, ^65^ However, the underlying mechanism remains unclear. A possible explanation is that these PD fragments promote GF interactions with its receptor complex. BFP‐1 (G^100^‐Q^114^) resides within the ^83^MLD^85^ and ^121^LQD^123^ cleavage sites.^64^ The sequence giving rise to the BFP‐2 fragment (V^140^‐R^154^) lies further downstream beyond the ^121^LQD^123^ cleavage site.^65^ Interestingly, cleavage at the ^107^YKA^109^ site would split the BFP‐1 peptide in two halves potentially leading to its inactivation. Our data suggest that MMP‐13 processes BMP‐7 PD first at the ^83^MLD^85^ site, followed by a secondary cleavage event at ^107^YKA^109^, which is succeeded by cleavage at the ^121^LQD^123^ site that will split BFP‐1 into two smaller fragments. MMP‐2 and MMP‐3 seem to follow a different cleavage sequence that does not utilize the ^107^YKA^109^ cleavage site (Figure 3A). This implies that despite most MMP cleavage events yielded BMP‐7 PD fragments of similar sizes, it is likely that PD processing by different MMPs yields PD fragments with different functional activity, which may add another layer of regulation of BMP activity in certain physiological conditions. For instance, in differentiation processes such as chondrogenic or osteogenic differentiation, where MMPs are known to play an important role,^66^, ^67^ BMP bioactivity may be further modulated by the generation of functionally active PD fragments.

The concept that proteases serve as specific activators of TGF‐β superfamily members through PD cleavage has been previously explored. TGF‐β‐1 PD could be cleaved in in vitro experiments by MMP‐2 and MMP‐9.^32^, ^59^ In addition, MMP‐14 activity, which depends on integrins, can promote TGF‐β‐1 activation.^33^ Furthermore, MMP cleavage of the TGF‐β PD was proposed as a mechanism to release TGF‐β GF from the ECM.^27^, ^31^ In addition, it could be shown that the metalloproteinase BMP‐1, a procollagen endopeptidase removing C‐terminal collagen propeptides, serves as GF activating enzyme by PD cleavage of several TGF‐β superfamily members. BMP‐1 is capable to process the PDs of GDF‐8, GDF‐11, and BMP‐10 PDs and thereby rendering the GF from latent to bioactive.^12^, ^35^, ^36^ Interestingly, in a previous cleavage screen with BMP‐1 and PDs of BMP‐4, ‐5, ‐7 no processing was observed,^12^ suggesting that different TGF‐β superfamily members are activated by different sets of metalloproteinases. This is probably reflecting the different cellular circumstances requiring activation of specific GFs. In the early stages of development where little ECM is present, active GFs are regulated by complexation to BMP antagonists such as chordin,^68^ which need to be also cleaved by BMP‐1/tolloid‐related metalloproteases to liberate the active GF.^68^, ^69^, ^70^ At later stages of development and certainly with the beginning of postnatal life when increasingly more ECM is present, PD‐ECM interactions facilitate GF targeting and sequestration. In this phase, MMPs start to regulate the bioavailability of tissue resident BMPs such as BMP‐7. However, for soluble BMPs such as BMP‐10 it could be shown that the PD complexation facilitates GF specificity toward cellular surfaces. While the BMP‐10 CPLX appeared to be latent toward C2C12 mouse myoblasts,^12^ it proved to be bioactive toward endothelial cells.^40^ BMP‐9 and ‐10 have been suggested to have redundant functions and also form hetero GF dimers.^71^, ^72^ Until now, the complex interplay of BMP‐9 and ‐10 functions in development and disease are not fully understood. As MMPs are also present in blood, controlled MMP cleavage of BMP‐9 and ‐10 PDs may represent a new mechanism to orchestrate their bioactivity under normal physiological or endothelial stress/disease conditions.

Our observation of BMP‐dependent induction of MMP expression (Figure 1B,C) was also found to be implicated in developmental or disease mechanisms accompanied by ECM degradation. For instance, BMP‐2 stimulation of the mouse myoblast cell line C2C12 initiated strong expression of MMP‐13.^73^ Also, BMP‐2 and ‐4 stimulation of primary human fibroblast led to MMP‐1, ‐2, ‐3, and ‐13 upregulation which was suggested to be a mechanism in melanoma invasion.^74^ Furthermore, other studies have shown that BMPs are drivers of tumor metastasis by inducing MMP expression and activity.^75^ For instance, BMP‐stimulated MMP‐2 and ‐9 activity was shown to be a relevant mechanism in breast cancer cell migration and invasion.^76^ Previous studies could associate BMP‐7 expression with early bone metastasis development in breast cancer.^77^, ^78^ MMP‐13 was described to be overexpressed at the tumor‐bone interface and abrogation of MMP‐13 in this area inhibited bone metastasis.^79^ As cancer often times recapitulates embryonic programs, it is not surprising that similar mechanisms occur during cartilage development. During chondrogenesis, BMPs control terminal differentiation where chondrocytes become hypertrophic and remove the collagen matrix through the upregulation of MMP‐13.^27^ Interestingly, during OA, chondrocytes in articular cartilage behave again as terminally differentiating chondrocytes. There, elevated BMP levels in damaged cartilage not only contribute to tissue repair by stimulating ECM synthesis but also promote cartilage degeneration by stimulating MMP‐13 expression.^80^

Overall, BMP‐stimulated MMP production appears to be an established mechanism during development and disease with the goal to rapidly remodel ECM architecture. Thereby, a fine‐tuned balance between BMP and MMP activity is crucial. In disease situations such as cancer or OA, small amounts of active BMP or MMP may initiate a vicious feed‐forward cycle where MMP‐mediated BMP release from ECM‐targeted pools further promotes MMP production ultimately resulting in severe ECM destruction. Similarly, in connective tissue disorders such as Marfan syndrome, failed BMP sequestration due to ECM deficiency may also trigger MMP‐mediated destruction cascade in tissues.

This study provides evidence for the existence of an MMP‐dependent mechanism for BMP activation from ECM‐targeted pools by PD cleavage. This knowledge may open up new therapeutic avenues, to impede pathomechanisms characterized by dysregulated BMP GF activity and ECM destruction.

## CONFLICT OF INTEREST

The authors declare no conflict of interest.

## AUTHOR CONTRIBUTIONS

G. Sengle designed the research. A.G. Furlan, C.E.S. Spanou, A.R.F. Godwin, A.P. Wohl, and L.M.A Zimmermann performed the research. T. Imhof and M. Koch contributed new reagents. A.G. Furlan, C.E.S. Spanou, A.R.F. Godwin, A.P. Wohl, L.M.A. Zimmermann, and C. Baldock analyzed the data. A.G. Furlan and G. Sengle wrote the manuscript.

## Supporting information

Fig S1

## References

[1] Shi Y , Massague J . Mechanisms of TGF‐beta signaling from cell membrane to the nucleus. Cell. 2003;113:685‐700. 12809600 10.1016/s0092-8674(03)00432-x

[2] Wu MY , Hill CS . Tgf‐beta superfamily signaling in embryonic development and homeostasis. Dev Cell. 2009;16:329‐343. 19289080 10.1016/j.devcel.2009.02.012

[3] Wang EA , Rosen V , D'Alessandro JS , et al. Recombinant human bone morphogenetic protein induces bone formation. Proc Natl Acad Sci U S A. 1990;87:2220‐2224. 2315314 10.1073/pnas.87.6.2220 PMC53658

[4] Gregory KE , Ono RN , Charbonneau NL , et al. The prodomain of BMP‐7 targets the BMP‐7 complex to the extracellular matrix. J Biol Chem. 2005;280:27970‐27980. 15929982 10.1074/jbc.M504270200

[5] Charbonneau NL , Ono RN , Corson GM , Keene DR , Sakai LY . Fine tuning of growth factor signals depends on fibrillin microfibril networks. Birth Defects Res C Embryo Today. 2004;72:37‐50. 15054903 10.1002/bdrc.20000

[6] Sengle G , Charbonneau NL , Ono RN , et al. Targeting of bone morphogenetic protein growth factor complexes to fibrillin. J Biol Chem. 2008;283:13874‐13888. 18339631 10.1074/jbc.M707820200 PMC2376219

[7] Tsuji K , Bandyopadhyay A , Harfe BD , et al. BMP2 activity, although dispensable for bone formation, is required for the initiation of fracture healing. Nat Genet. 2006;38:1424‐1429. 17099713 10.1038/ng1916

[8] Paralkar VM , Weeks BS , Yu YM , Kleinman HK , Reddi AH . Recombinant human bone morphogenetic protein 2B stimulates PC12 cell differentiation: potentiation and binding to type IV collagen. J Cell Biol. 1992;119:1721‐1728. 1469059 10.1083/jcb.119.6.1721 PMC2289768

[9] Suzawa M , Takeuchi Y , Fukumoto S , et al. Extracellular matrix‐associated bone morphogenetic proteins are essential for differentiation of murine osteoblastic cells in vitro. Endocrinology. 1999;140:2125‐2133. 10218963 10.1210/endo.140.5.6704

[10] Sieron AL , Louneva N , Fertala A . Site‐specific interaction of bone morphogenetic protein 2 with procollagen II. Cytokine. 2002;18:214‐221. 12126644 10.1006/cyto.2002.1035

[11] Wang X , Harris RE , Bayston LJ , Ashe HL . Type IV collagens regulate BMP signalling in Drosophila. Nature. 2008;455:72‐77. 18701888 10.1038/nature07214

[12] Sengle G , Ono RN , Sasaki T , Sakai LY . Prodomains of transforming growth factor beta (TGFbeta) superfamily members specify different functions: extracellular matrix interactions and growth factor bioavailability. J Biol Chem. 2011;286:5087‐5099. 21135108 10.1074/jbc.M110.188615 PMC3037620

[13] Sakai LY , Keene DR , Engvall E . Fibrillin, a new 350‐kD glycoprotein, is a component of extracellular microfibrils. J Cell Biol. 1986;103:2499‐2509. 3536967 10.1083/jcb.103.6.2499 PMC2114568

[14] Sakai LY , Keene DR , Renard M , De Backer J . FBN1: the disease‐causing gene for Marfan syndrome and other genetic disorders. Gene. 2016;591:279‐291. 27437668 10.1016/j.gene.2016.07.033 PMC6639799

[15] Loeys BL , Matthys DM , de Paepe AM . Genetic fibrillinopathies: new insights in molecular diagnosis and clinical management. Acta Clin Belg. 2003;58:3‐11. 12723256 10.1179/acb.2003.58.1.001

[16] Nistala H , Lee‐Arteaga S , Smaldone S , et al. Fibrillin‐1 and ‐2 differentially modulate endogenous TGF‐beta and BMP bioavailability during bone formation. J Cell Biol. 2010;190:1107‐1121. 20855508 10.1083/jcb.201003089 PMC3101602

[17] Arteaga‐Solis E , Gayraud B , Lee SY , Shum L , Sakai L , Ramirez F . Regulation of limb patterning by extracellular microfibrils. J Cell Biol. 2001;154:275‐281. 11470817 10.1083/jcb.200105046 PMC2150751

[18] Sengle G , Carlberg V , Tufa SF , et al. Abnormal activation of BMP signaling causes myopathy in Fbn2 null mice. PLoS Genet. 2015;11:e1005340. 26114882 10.1371/journal.pgen.1005340 PMC4482570

[19] Ramirez F , Rifkin DB . Extracellular microfibrils: contextual platforms for TGFbeta and BMP signaling. Curr Opin Cell Biol. 2009;21:616‐622. 19525102 10.1016/j.ceb.2009.05.005 PMC2767232

[20] Sengle G , Sakai LY . The fibrillin microfibril scaffold: a niche for growth factors and mechanosensation? Matrix Biol. 2015;47:3‐12. 25957947 10.1016/j.matbio.2015.05.002

[21] Miyazono K , Hellman U , Wernstedt C , Heldin CH . Latent high molecular weight complex of transforming growth factor beta 1. Purification from human platelets and structural characterization. J Biol Chem. 1988;263:6407‐6415. 3162913

[22] Unsold C , Hyytiainen M , Bruckner‐Tuderman L , Keski‐Oja J . Latent TGF‐beta binding protein LTBP‐1 contains three potential extracellular matrix interacting domains. J Cell Sci. 2001;114:187‐197. 11112702 10.1242/jcs.114.1.187

[23] Saharinen J , Keski‐Oja J . Specific sequence motif of 8‐Cys repeats of TGF‐beta binding proteins, LTBPs, creates a hydrophobic interaction surface for binding of small latent TGF‐beta. Mol Biol Cell. 2000;11:2691‐2704. 10930463 10.1091/mbc.11.8.2691 PMC14949

[24] Dallas SL , Sivakumar P , Jones CJ , et al. Fibronectin regulates latent transforming growth factor‐beta (TGF beta) by controlling matrix assembly of latent TGF beta‐binding protein‐1. J Biol Chem. 2005;280:18871‐18880. 15677465 10.1074/jbc.M410762200

[25] Isogai Z , Ono RN , Ushiro S , et al. Latent transforming growth factor beta‐binding protein 1 interacts with fibrillin and is a microfibril‐associated protein. J Biol Chem. 2003;278:2750‐2757. 12429738 10.1074/jbc.M209256200

[26] Ono RN , Sengle G , Charbonneau NL , et al. Latent transforming growth factor beta‐binding proteins and fibulins compete for fibrillin‐1 and exhibit exquisite specificities in binding sites. J Biol Chem. 2009;284:16872‐16881. 19349279 10.1074/jbc.M809348200 PMC2719323

[27] Dangelo M , Sarment DP , Billings PC , Pacifici M . Activation of transforming growth factor beta in chondrocytes undergoing endochondral ossification. J Bone Miner Res. 2001;16:2339‐2347. 11760850 10.1359/jbmr.2001.16.12.2339

[28] Fontana L , Chen Y , Prijatelj P , et al. Fibronectin is required for integrin alphavbeta6‐mediated activation of latent TGF‐beta complexes containing LTBP‐1. FASEB J. 2005;19:1798‐1808. 16260650 10.1096/fj.05-4134com

[29] Shi M , Zhu J , Wang R , et al. Latent TGF‐beta structure and activation. Nature. 2011;474:343‐349. 21677751 10.1038/nature10152 PMC4717672

[30] Buscemi L , Ramonet D , Klingberg F , et al. The single‐molecule mechanics of the latent TGF‐beta1 complex. Curr Biol. 2011;21:2046‐2054. 22169532 10.1016/j.cub.2011.11.037

[31] Jenkins G . The role of proteases in transforming growth factor‐beta activation. Int J Biochem Cell Biol. 2008;40:1068‐1078. 18243766 10.1016/j.biocel.2007.11.026

[32] Yu Q , Stamenkovic I . Cell surface‐localized matrix metalloproteinase‐9 proteolytically activates TGF‐beta and promotes tumor invasion and angiogenesis. Genes Dev. 2000;14:163‐176. 10652271 PMC316345

[33] Mu D , Cambier S , Fjellbirkeland L , et al. The integrin alpha(v)beta8 mediates epithelial homeostasis through MT1‐MMP‐dependent activation of TGF‐beta1. J Cell Biol. 2002;157:493‐507. 11970960 10.1083/jcb.200109100 PMC2173277

[34] Ge G , Greenspan DS . BMP1 controls TGFbeta1 activation via cleavage of latent TGFbeta‐binding protein. J Cell Biol. 2006;175:111‐120. 17015622 10.1083/jcb.200606058 PMC2064503

[35] Wolfman NM , McPherron AC , Pappano WN , et al. Activation of latent myostatin by the BMP‐1/tolloid family of metalloproteinases. Proc Natl Acad Sci U S A. 2003;100:15842‐15846. 14671324 10.1073/pnas.2534946100 PMC307655

[36] Ge G , Hopkins DR , Ho WB , Greenspan DS . GDF11 forms a bone morphogenetic protein 1‐activated latent complex that can modulate nerve growth factor‐induced differentiation of PC12 cells. Mol Cell Biol. 2005;25:5846‐5858. 15988002 10.1128/MCB.25.14.5846-5858.2005 PMC1168807

[37] Le VQ , Iacob RE , Tian Y , et al. Tolloid cleavage activates latent GDF8 by priming the pro‐complex for dissociation. EMBO J. 2018;37:384‐397. 29343545 10.15252/embj.201797931 PMC5793799

[38] Brown MA , Zhao Q , Baker KA , et al. Crystal structure of BMP‐9 and functional interactions with pro‐region and receptors. J Biol Chem. 2005;280:25111‐25118. 15851468 10.1074/jbc.M503328200

[39] Sengle G , Ono RN , Lyons KM , Bachinger HP , Sakai LY . A new model for growth factor activation: type II receptors compete with the prodomain for BMP‐7. J Mol Biol. 2008;381:1025‐1039. 18621057 10.1016/j.jmb.2008.06.074 PMC2705212

[40] Jiang H , Salmon RM , Upton PD , et al. The prodomain‐bound form of bone morphogenetic protein 10 is biologically active on endothelial cells. J Biol Chem. 2016;291:2954‐2966. 26631724 10.1074/jbc.M115.683292 PMC4742757

[41] Kienast Y , Jucknischke U , Scheiblich S , et al. Rapid activation of bone morphogenic protein 9 by receptor‐mediated displacement of Pro‐domains. J Biol Chem. 2016;291:3395‐3410. 26677222 10.1074/jbc.M115.680009 PMC4751383

[42] Wohl AP , Troilo H , Collins RF , Baldock C , Sengle G . extracellular regulation of bone morphogenetic protein activity by the microfibril component fibrillin‐1. J Biol Chem. 2016;291:12732‐12746. 27059954 10.1074/jbc.M115.704734 PMC4933460

[43] Hiepen C , Jatzlau J , Hildebrandt S , et al. BMPR2 acts as a gatekeeper to protect endothelial cells from increased TGFbeta responses and altered cell mechanics. PLoS Biol. 2019;17:e3000557. 31826007 10.1371/journal.pbio.3000557 PMC6927666

[44] Lichti U , Anders J , Yuspa SH . Isolation and short‐term culture of primary keratinocytes, hair follicle populations and dermal cells from newborn mice and keratinocytes from adult mice for in vitro analysis and for grafting to immunodeficient mice. Nat Protoc. 2008;3:799‐810. 18451788 10.1038/nprot.2008.50 PMC6299324

[45] Livak KJ , Schmittgen TD . Analysis of relative gene expression data using real‐time quantitative PCR and the 2(‐Delta Delta C(T)) Method. Methods. 2001;25:402‐408. 11846609 10.1006/meth.2001.1262

[46] Kuo CL , Isogai Z , Keene DR , et al. Effects of fibrillin‐1 degradation on microfibril ultrastructure. J Biol Chem. 2007;282:4007‐4020. 17158461 10.1074/jbc.M606370200

[47] Koch M , Veit G , Stricker S , et al. Expression of type XXIII collagen mRNA and protein. J Biol Chem. 2006;281:21546‐21557. 16728390 10.1074/jbc.M604131200

[48] Hedman K , Kurkinen M , Alitalo K , Vaheri A , Johansson S , Hook M . Isolation of the pericellular matrix of human fibroblast cultures. J Cell Biol. 1979;81:83‐91. 383722 10.1083/jcb.81.1.83 PMC2111519

[49] Berry R , Jowitt TA , Ferrand J , et al. Role of dimerization and substrate exclusion in the regulation of bone morphogenetic protein‐1 and mammalian tolloid. Proc Natl Acad Sci U S A. 2009;106:8561‐8566. 19429706 10.1073/pnas.0812178106 PMC2689009

[50] Kimanius D , Forsberg BO , Scheres SHW , Lindahl E . Accelerated cryo‐EM structure determination with parallelisation using GPUs in RELION‐2. eLife. 2016;5:e18722. 27845625 10.7554/eLife.18722 PMC5310839

[51] Tang G , Peng L , Baldwin PR , et al. EMAN2: an extensible image processing suite for electron microscopy. J Struct Biol. 2007;157:38‐46. 16859925 10.1016/j.jsb.2006.05.009

[52] Sali A , Blundell TL . Comparative protein modelling by satisfaction of spatial restraints. J Mol Biol. 1993;234:779‐815. 8254673 10.1006/jmbi.1993.1626

[53] Pettersen EF , Goddard TD , Huang CC , et al. UCSF Chimera–a visualization system for exploratory research and analysis. J Comput Chem. 2004;25:1605‐1612. 15264254 10.1002/jcc.20084

[54] Kozakov D , Hall DR , Xia B , et al. The ClusPro web server for protein‐protein docking. Nat Protoc. 2017;12:255‐278. 28079879 10.1038/nprot.2016.169 PMC5540229

[55] Liu Y , Li Y , Li N , et al. TGF‐beta1 promotes scar fibroblasts proliferation and transdifferentiation via up‐regulating MicroRNA‐21. Sci Rep. 2016;6:32231. 27554193 10.1038/srep32231 PMC4995376

[56] Nagase H , Visse R , Murphy G . Structure and function of matrix metalloproteinases and TIMPs. Cardiovasc Res. 2006;69:562‐573. 16405877 10.1016/j.cardiores.2005.12.002

[57] Kumar S , Ratnikov BI , Kazanov MD , Smith JW , Cieplak P . CleavPredict: a platform for reasoning about matrix metalloproteinases proteolytic events. PLoS One. 2015;10:e0127877. 25996941 10.1371/journal.pone.0127877 PMC4440711

[58] Eckhard U , Huesgen PF , Schilling O , et al. Active site specificity profiling of the matrix metalloproteinase family: proteomic identification of 4300 cleavage sites by nine MMPs explored with structural and synthetic peptide cleavage analyses. Matrix Biol. 2016;49:37‐60. 26407638 10.1016/j.matbio.2015.09.003

[59] Wang M , Zhao D , Spinetti G , et al. Matrix metalloproteinase 2 activation of transforming growth factor‐beta1 (TGF‐beta1) and TGF‐beta1‐type II receptor signaling within the aged arterial wall. Arterioscler Thromb Vasc Biol. 2006;26:1503‐1509. 16690877 10.1161/01.ATV.0000225777.58488.f2

[60] Stura EA , Visse R , Cuniasse P , Dive V , Nagase H . Crystal structure of full‐length human collagenase 3 (MMP‐13) with peptides in the active site defines exosites in the catalytic domain. FASEB J. 2013;27:4395‐4405. 23913860 10.1096/fj.13-233601 PMC3804752

[61] Lyons RM , Gentry LE , Purchio AF , Moses HL . Mechanism of activation of latent recombinant transforming growth factor beta 1 by plasmin. J Cell Biol. 1990;110:1361‐1367. 2139036 10.1083/jcb.110.4.1361 PMC2116088

[62] Ribeiro SM , Poczatek M , Schultz‐Cherry S , Villain M , Murphy‐Ullrich JE . The activation sequence of thrombospondin‐1 interacts with the latency‐associated peptide to regulate activation of latent transforming growth factor‐beta. J Biol Chem. 1999;274:13586‐13593. 10224129 10.1074/jbc.274.19.13586

[63] Park HI , Lee S , Ullah A , Cao Q , Sang QX . Effects of detergents on catalytic activity of human endometase/matrilysin 2, a putative cancer biomarker. Anal Biochem. 2010;396:262‐268. 19818727 10.1016/j.ab.2009.10.005 PMC2801054

[64] Kim HK , Kim JH , Park DS , et al. Osteogenesis induced by a bone forming peptide from the prodomain region of BMP‐7. Biomaterials. 2012;33:7057‐7063. 22795855 10.1016/j.biomaterials.2012.06.036

[65] Kim HK , Lee JS , Kim JH , et al. Bone‐forming peptide‐2 derived from BMP‐7 enhances osteoblast differentiation from multipotent bone marrow stromal cells and bone formation. Exp Mol Med. 2017;49:e328. 28496198 10.1038/emm.2017.40 PMC5454442

[66] Assis‐Ribas T , Forni MF , Winnischofer SMB , Sogayar MC , Trombetta‐Lima M . Extracellular matrix dynamics during mesenchymal stem cells differentiation. Dev Biol. 2018;437:63‐74. 29544769 10.1016/j.ydbio.2018.03.002

[67] Almalki SG , Agrawal DK . Effects of matrix metalloproteinases on the fate of mesenchymal stem cells. Stem Cell Res Ther. 2016;7:129. 27612636 10.1186/s13287-016-0393-1 PMC5016871

[68] Troilo H , Zuk AV , Tunnicliffe RB , et al. Nanoscale structure of the BMP antagonist chordin supports cooperative BMP binding. Proc Natl Acad Sci U S A. 2014;111:13063‐13068. 25157165 10.1073/pnas.1404166111 PMC4246984

[69] Scott IC , Blitz IL , Pappano WN , et al. Mammalian BMP‐1/Tolloid‐related metalloproteinases, including novel family member mammalian Tolloid‐like 2, have differential enzymatic activities and distributions of expression relevant to patterning and skeletogenesis. Dev Biol. 1999;213:283‐300. 10479448 10.1006/dbio.1999.9383

[70] Piccolo S , Agius E , Lu B , Goodman S , Dale L , De Robertis EM . Cleavage of Chordin by Xolloid metalloprotease suggests a role for proteolytic processing in the regulation of Spemann organizer activity. Cell. 1997;91:407‐416. 9363949 10.1016/s0092-8674(00)80424-9 PMC3070600

[71] Ricard N , Ciais D , Levet S , et al. BMP9 and BMP10 are critical for postnatal retinal vascular remodeling. Blood. 2012;119:6162‐6171. 22566602 10.1182/blood-2012-01-407593 PMC3383024

[72] Tillet E , Ouarne M , Desroches‐Castan A , et al. A heterodimer formed by bone morphogenetic protein 9 (BMP9) and BMP10 provides most BMP biological activity in plasma. J Biol Chem. 2018;293:10963‐10974. 29789425 10.1074/jbc.RA118.002968 PMC6052235

[73] Nakashima A , Tamura M . Regulation of matrix metalloproteinase‐13 and tissue inhibitor of matrix metalloproteinase‐1 gene expression by WNT3A and bone morphogenetic protein‐2 in osteoblastic differentiation. Front Biosci. 2006;11:1667‐1678. 16368545 10.2741/1912

[74] Rothhammer T , Braig S , Bosserhoff AK . Bone morphogenetic proteins induce expression of metalloproteinases in melanoma cells and fibroblasts. Eur J Cancer. 2008;44:2526‐2534. 18774289 10.1016/j.ejca.2008.07.029

[75] Bach DH , Park HJ , Lee SK . The dual role of bone morphogenetic proteins in cancer. Mol Ther Oncolytics. 2018;8:1‐13. 29234727 10.1016/j.omto.2017.10.002 PMC5723373

[76] Cyr‐Depauw C , Northey JJ , Tabaries S , et al. Chordin‐Like 1 suppresses bone morphogenetic protein 4‐induced breast cancer cell migration and invasion. Mol Cell Biol. 2016;36:1509‐1525. 26976638 10.1128/MCB.00600-15 PMC4859683

[77] Alarmo EL , Parssinen J , Ketolainen JM , Savinainen K , Karhu R , Kallioniemi A . BMP7 influences proliferation, migration, and invasion of breast cancer cells. Cancer Lett. 2009;275:35‐43. 18980801 10.1016/j.canlet.2008.09.028

[78] Sakai H , Furihata M , Matsuda C , et al. Augmented autocrine bone morphogenic protein (BMP) 7 signaling increases the metastatic potential of mouse breast cancer cells. Clin Exp Metastasis. 2012;29:327‐338. 22274590 10.1007/s10585-012-9453-9

[79] Nannuru KC , Futakuchi M , Varney ML , Vincent TM , Marcusson EG , Singh RK . Matrix metalloproteinase (MMP)‐13 regulates mammary tumor‐induced osteolysis by activating MMP9 and transforming growth factor‐beta signaling at the tumor‐bone interface. Cancer Res. 2010;70:3494‐3504. 20406980 10.1158/0008-5472.CAN-09-3251 PMC2862120

[80] van der Kraan PM , Blaney Davidson EN , van den Berg WB . Bone morphogenetic proteins and articular cartilage: to serve and protect or a wolf in sheep clothing's? Osteoarthritis Cartilage. 2010;18:735‐741. 20211748 10.1016/j.joca.2010.03.001

