# Supplementary material for: A new MMP‐mediated prodomain cleavage mechanism to activate bone morphogenetic proteins from the extracellular matrix
Source: FASEB J. 2021 Feb 25;35(3):e21353. doi: 10.1096/fj.202001264R (PMC12266326; doi:10.1096/fj.202001264R)
Supplement: Supplementary file 1 — Fig S1 [file FSB2-35-e21353-s001.pdf]

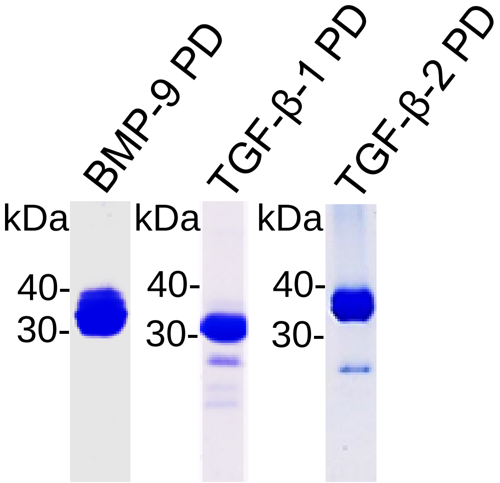

Supplementary Figure S1: Affinity purification of recombinant TGF-β superfamily PDs used in this study. Coomassie Brilliant Blue-stained SDS-PAGE quality control gels of recombinantly expressed and affinity-purified PDs.

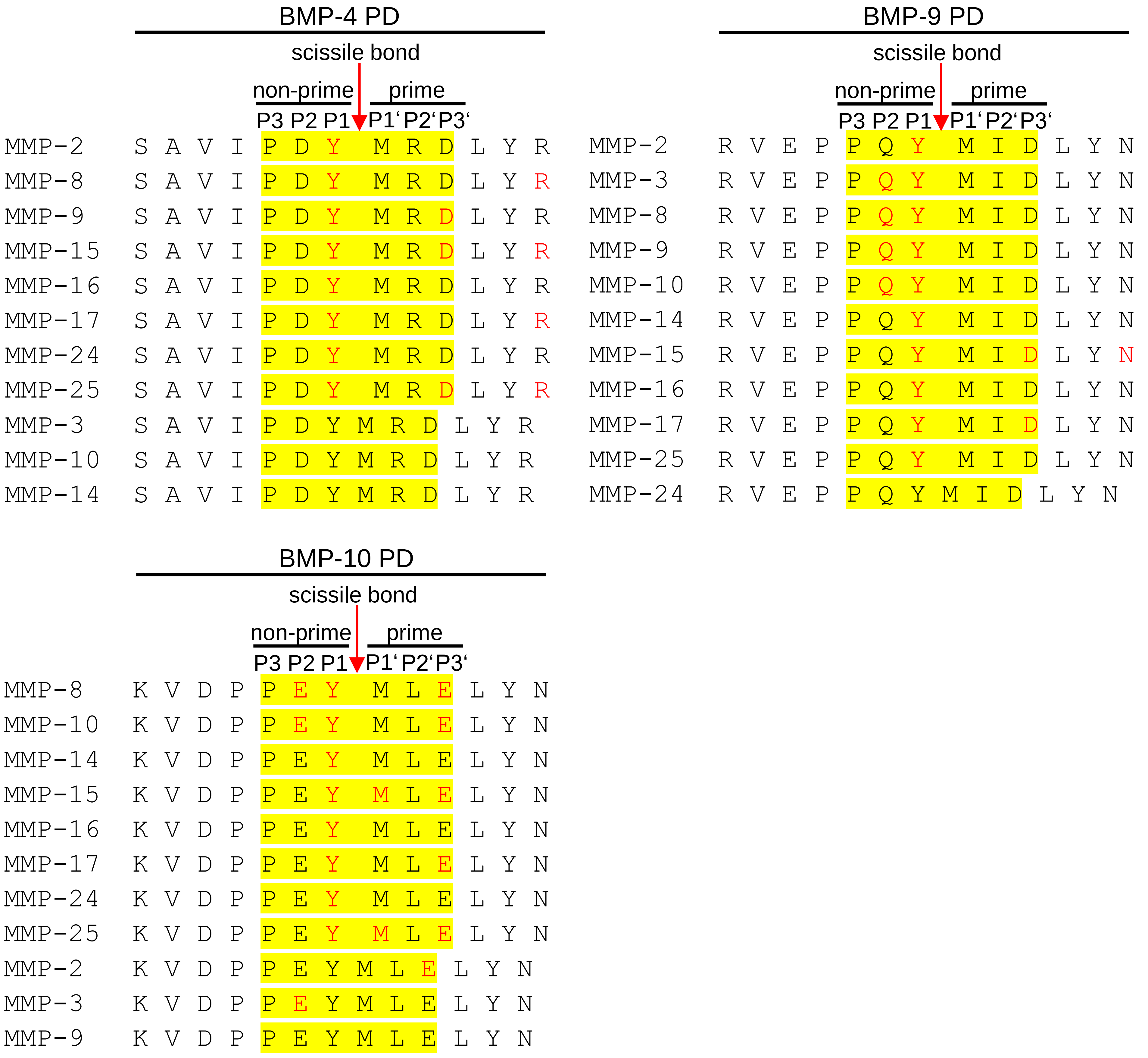

Supplementary Figure S2: Predicted MMP cleavage sites in BMP-4, -9, and 10 PDs using the CleavPredict platform. P1 cleavage positions before the predicted scissile bond (red arrow) are indicated in red**.** Six residues of each predicted cleavage site in non-prime (P3, P2, P1) and prime (P1', P2', P3') region are marked in yellow.

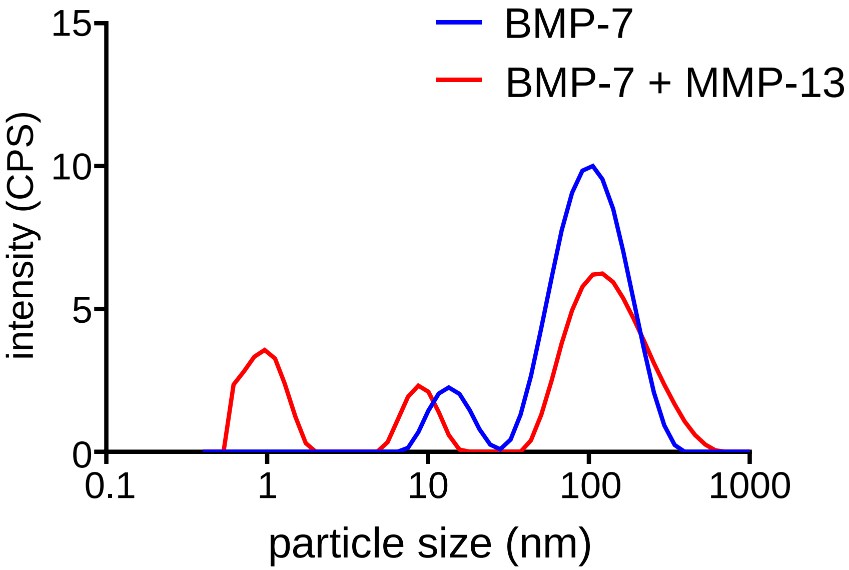

Supplementary Figure S3: Dynamic light scattering (DLS) of BMP-7 CPLX after MMP-13 cleavage. Intensity size distribution of BMP-7 CPLX incubated in the presence or absence of MMP-13. The y-axis represents counts per second (CPS) and the x-axis represents particle size in nm.
